# Advances of Hydroxyapatite Nanoparticles in Dental Implant Applications

**DOI:** 10.1016/j.identj.2024.11.020

**Published:** 2025-01-10

**Authors:** Md. Aminul Islam, Nayem Hossain, Sumaya Hossain, Fardin Khan, Saniya Hossain, Md. Mostafizur Rahman Arup, Mohammad Asaduzzaman Chowdhury, Md. Majibur Rahman

**Affiliations:** a Department of Mechanical Engineering, International University of Business Agriculture and Technology, Dhaka, Dhaka, Bangladesh; b Department of Pharmacy, Primeasia University, Dhaka, Dhaka, Bangladesh; c Department of Microbiology, Jashore University of Science and Technology, Jessore, Jessore, Bangladesh; d Department of Mechanical Engineering, Dhaka University of Engineering and Technology, Gazipur, Gazipur, Bangladesh; e Department of Microbiology, University of Dhaka, Dhaka, Dhaka, Bangladesh

**Keywords:** Hydroxyapatite, Implant, Bone regeneration, Biodegradation, Dental treatments

## Abstract

Hydroxyapatite nanoparticles (HANPs) are becoming increasingly crucial in dental implant applications as they are highly compatible with biological systems, actively support biological processes, and closely resemble bone minerals. This review covers the latest progress in how HANPs are made, studied, and used in dentistry. It looks at critical methods for creating HANPs, such as sol-gel, microwave hydrothermal synthesis, and biomimetic approaches, and how they affect the particles' size, structure, and activity. The green synthesis method illustrated a new door to synthesize HAp for maintaining biocompatibilityand increasing antibacterial properties. The review also explores how HANPs improve the integration of implants with bone, support bone growth, and help treat sensitive teeth based on various laboratory and clinical studies. The usage of HAp in dentin and enamel shows higher potentiality through FTIR, XPS, XRD, EDS, etc., for mechanical stability and biological balance compared to natural teeth. Additionally, the use of HANPs in dental products like toothpaste and mouthwash is discussed, highlighting its potential to help rebuild tooth enamel and fight bacteria. There are some challenges for long-term usage against oral bacteria, but doping with inorganic materials, like Zn, has already solved this periodontal problem. Much more research is still essential to estimate the fabrication variation based on patient problems and characteristics. Still, it has favorable outcomes regarding its bioactive nature and antimicrobial properties. Due to their compatibility with biological tissues and ability to support bone growth, HANPs hold great promise for advancing dental materials and implant technology, potentially leading to better dental care and patient outcomes.

## Introduction

Particulate dispersion or solid particles with a size between 10 and 1000 nm are referred to as nanoparticles.^1^ They are mysterious because a significant portion of their atoms are at or close to surfaces, affecting the particle's atomic, electronic, and magnetic structures, physical and chemical properties, and reactivity compared to the bulk material.^2^ Many medical specialties, including cancer and cardiovascular health, have included nanoparticles. The care of these patients may benefit from the application of nanoparticles to improve the identification of biomarkers, molecular diagnostics, medication development, and drug delivery.^3^

Apatite is a flexible term that describes a range of crystalline minerals the formula can represent. $M_{10}{\left(ZO_{4}\right)}_{6}X_{2}$, with each component (M, $ZO_{4}$, And X) substitutable by a diverse range of ions, as listed in Table 1. Calcium phosphate apatite is the most commonly occurring type of apatite in nature, with $Ca^{2+}$ and $PO_{4}^{3-}$ serving as the M and $ZO_{4}$Components respectively. This mineral is known as Hydroxyapatite (HA) when $OH^{-}$ represents the X component in its chemical formula, $Ca_{10}{\left(PO_{4}\right)}_{6}{\left(OH\right)}_{2}$, Which has a stoichiometric Ca/P molar ratio of 1.67.^4^, ^5^, ^6^ Hydroxyapatite is a calcium(C), phosphorus(P), and hydroxide ($OH^{-}$) Compound, which is the main mineral component in teeth and bones. It contributes to around 65% of bone structure, providing necessary rigidity and strength to bones and teeth.^7^ About 65 percent of the bone's composition is HA, a needle-shaped substance with dimensions of 60 nm in length and 5-20 nm in width.^8^ Due to its resemblance to the body's complex tissues, Hydroxyapatite has remarkable biocompatibility and bioactivity features in bone cells and tissues.^9^ In addition, Hydroxyapatite is found in the bone as nanoscale plates or needles that are 40-60 nm long, 5-20 nm broad, and 1.5-5 nm thick; advancements in nanotechnologies have made it possible to create Nano hydroxyapatite (HANPs) materials that more closely resemble genuine bone.^10^

**Table 1:** Major ions that can be part of apatite, $M_{10}{\left(ZO_{4}\right)}_{6}X_{2}$.

Component	Ions
M	$Ca^{2+},\;Mg^{2+},\;Sr^{2+},\;Ba^{2+},\;Mn^{2+},\;Fe^{2+},\;Fe^{2+},\;Zn^{2+},\;Cd^{2+},\;Pb^{2+},H^{+},\;Na^{+},\;K^{+},\;Al^{3+},\;etc$
$ZO_{4}$	$PO_{4}^{3-},\;AsO_{4}^{3-},\;VO_{4}^{3-},\;SO_{4}^{3-},\;CO_{3}^{2-},\;SiO_{4}^{3-},\;etc$
X	$OH^{-},\;F^{-},\;Cl^{-},\;Br^{-},\;O^{2-},\;CO_{3}^{2-},\;etc$

Hydroxyapatite crystals dominate 2 of its essential layers: enamel and dentin. Enamel, the outermost layer, comprises 96% inorganic hydroxyapatite crystals, some organic constituents like proteins and lipids, and 4% water. Meanwhile, the underlying dentin layer shall comprise 70% inorganic hydroxyapatite matrix, 20% organic matrix, and 10% water.^7^^,^^11^ X-ray Diffraction (XRD) analysis has determined that the typical size of hydroxyapatite crystallites in enamel is 48 to 78 nm.^8^ HA crystallites have distinct shapes and arrangements in enamel and mature dentine. In enamel, they form hexagonal rods with a diameter of 4 µm, while in mature dentine, they become flattened plates measuring 60-70 nm in length, 3-4 nm in thickness, and 20-30 nm in width.^11^

Hydroxyapatite (HAp) improves the efficiency of the controlled drug delivery mechanism and has great promise for treating several complex medical issues. Various synthesis methods have been adopted to fabricate nanostructured hydroxyapatite (HANPs) worldwide. Efforts have been made to control their geometry, crystallinity, size, stoichiometry, and degree of particle agglomeration (for different applications) by employing new routes or modifying pre-existing synthesis methods.^12^ Additionally, some other molecules, like tantalum and strontium, can be doped with HAp for coating, emphasizing surface modification. This doping also creates a porous structure to enhance the growth of cells in modified surface texture for dental implants.^13^

Recently, Hydroxyapatite nanoparticles started to be used as an oral care ingredient in commercially available toothpaste, mouthwashes, and other dental products.^14^, ^15^ The oral cavity contains teeth comprising several components, including pulp, cementum, dentin, enamel, and periodontal ligament. The purpose of teeth is to chop and compress food so that it's simpler to swallow and digest.^16^ Research shows that infected teeth experience more pressure rather than healthy teeth. For this reason, cavities during implants cause complicated problems.^17^ However, among the many available alternatives, 1 of the most often used techniques is coating dental implants with a hydroxyapatite layer due to its exceptional biocompatibility and osteoconductive activity.^18^ The increased osseous tissue integration to coated implant surfaces is critical to the positive results of hydroxyapatite coatings. Due to its parallel structure to bone minerals, this substance was intended to be a bone replacement product.^19^ Numerous studies have examined the possibility of using systems reinforced with HA NPs to successfully treat Dentinal Hypersensitivity (DH) due to their remineralization characteristics.^20^ Vjayasankari et al. (2019) aimed to evaluate the remineralization potential of an experimental nano-HA paste on artificial carious lesions by using Scanning Electron Microscopy (SEM) with Energy-Dispersive X-ray (EDX). All 4 pastes tested (1% experimental nano-HA paste, 10% nano-HA experimental paste, 1% commercially available nano-HA paste, and a Casein Phosphopeptide–Amorphous Calcium Phosphate (CPP–ACP)) showed significant changes in calcium and phosphorus weight percentages after remineralization. The 10% nano-HA experimental paste determined the highest mean value of calcium and phosphorus weight percentage. After remineralization, both 10% experimental nano-HA paste and CPP–ACP paste showed favorable enamel surface changes in SEM analysis.^21^ The placement of dental implants is a commonly observed procedure, as evidenced by an estimated annual insertion of 100,000-300,000 dental implants. This number is comparable to the yearly placement of artificial hip and knee joints, underscoring the importance of dental implants as a significant and relevant option for dental restoration.^22^, ^23^

It has been the subject of numerous studies due to its exceptional biocompatibility and ability to promote bone growth. As a result, this bioceramics has been extensively investigated for various biomedical applications, including implant coatings, bone scaffolds, bone fillers, and drug delivery systems.^7^^,^^24^, ^25^, ^26^ Tooth loss is prevalent due to various factors, such as disease or trauma. As a result, dental implants have a rich and diverse history of being used to support the replacement of missing teeth.^27^, ^28^, ^29^, ^30^, ^31^, ^32^ Based on data released by the American Association of Oral and Maxillofacial Surgeons, a notable proportion of individuals aged 35-44, comprising around 69%, have suffered from the loss of at least 1 permanent tooth due to a variety of reasons, such as gum disease, tooth decay, root canal failure, or accidents. Moreover, the same report highlights that roughly 26% of adults aged 74 or older have lost all their permanent teeth.^27^^,^^33^ The field of dental implant research has seen a notable surge in the exploration of various designs, materials, and techniques over the past few years, with expectations for continued expansion in the future.^27^, ^28^^,^^31^^,^^34^

Figure 1.

**Fig. 1:**
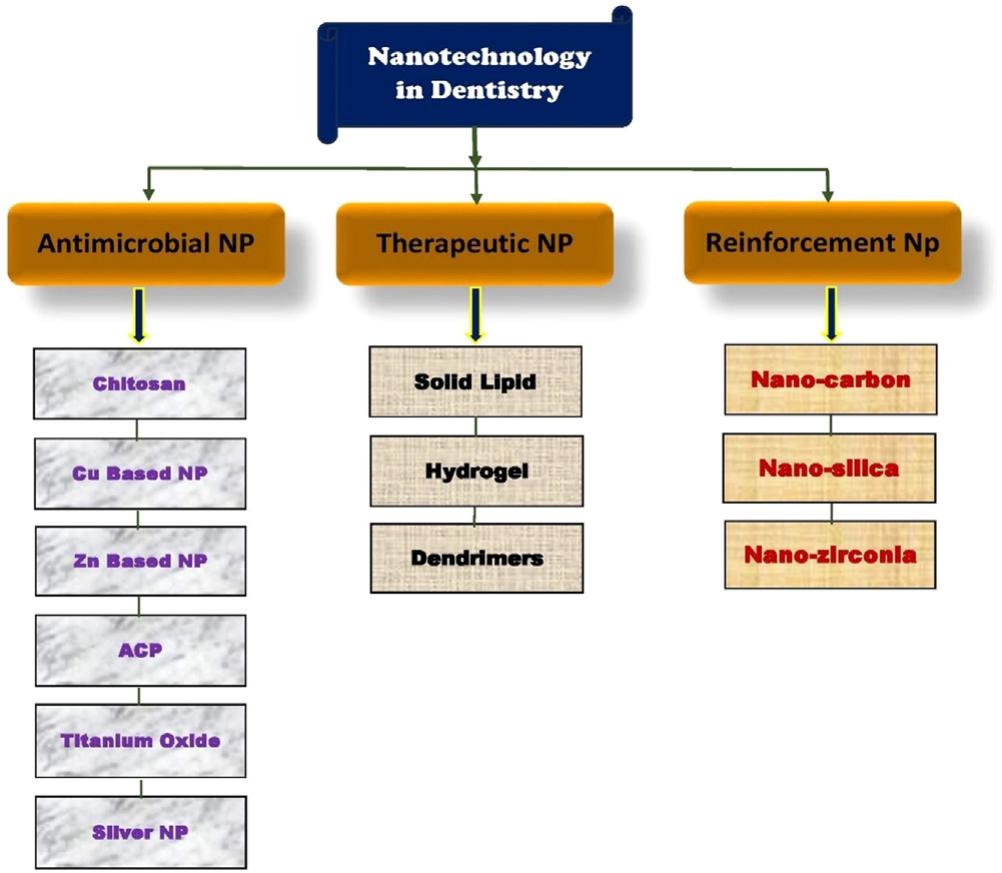
Dental Application of Nanoparticle.^35^

HA crystals found in natural bone are similar in size and characteristics to nanoscale HA.^36^ The usage of Hydroxyapatite materials is predicted to be able to stop infection from being the reason for early or late dental implant failure.^10^ The field of dental implant research has seen a notable surge in the exploration of various designs, materials, and techniques over the past few years, with expectations for continued expansion in the future.^22^^,^^37^, ^38^, ^39^ The research of hydroxyapatite nanoparticles (HA NPs) in dental implant applications is justified by the fact that have a significant potential to influence dental care. Because HANPs are biocompatible, they closely resemble the composition and characteristics of dental enamel and genuine bone. The ability to promote osseointegration, bone regeneration, and fight bacterial infections makes them excellent dental materials. It investigates novel synthesis techniques and creative dental applications to enhance patient results. The study discussed in this paper aims to improve implant success rates and oral health by meeting the current demands for innovative, biocompatible materials in healthcare.^40^, ^41^

This work's uniqueness stems from its thorough but scientific approach to producing, functionalizing, and clinical advantages of hydroxyapatite nanoparticles (HANPs) for dental implants. It combines insights from recent experimental work on remineralization and antibacterial qualities with novel production processes, including sol-gel and microwave hydrothermal synthesis, for clean and efficient implant osseointegration and biocompatibility. The creative development of this increased emphasis on HANPs for improved dental material improvement mirrors advancements in dental materials that improve patient outcomes.^42^, ^43^ However, this review sheds some light on Hydroxyapatite Nanoparticles in Dental Implant Applications.

## Hydroxyapatite use in dentistry

**Fig. 2:**
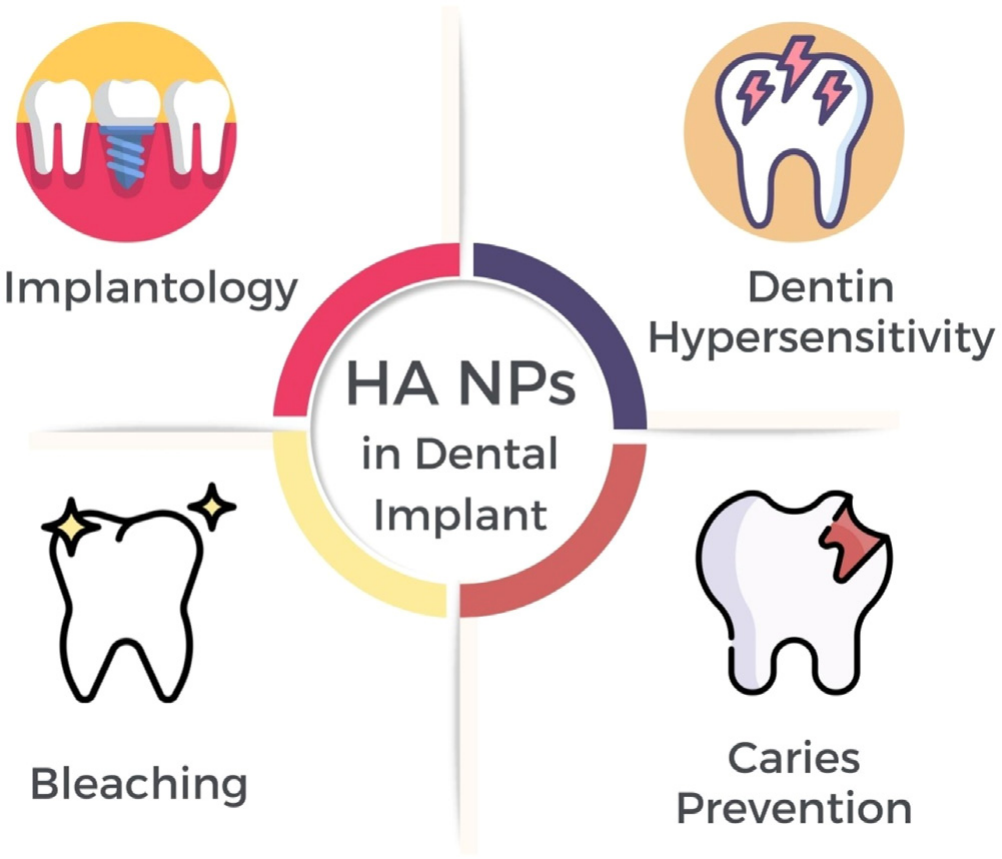
HANPs in dental implants.

Nano-sized particles have found significant utility in dentistry, particularly as fillers for developing nanocomposites.^44^ HANPs have various applications in dental treatment for different purposes, which have already been implemented in the medical field. Figure 2 depicts the application utilized in dental science at this time.

Table 2 summarizes recent research findings that strongly support nanohydroxyapatite utilization in diverse dental practice areas, including dental implantology, dentin hypersensitivity management, bleaching, dental caries prevention, and tooth restoration. These investigations underscore the effectiveness and adaptability of nanohydroxyapatite in various dental applications, underscoring its capacity to drive advancements in dental care and HANP since patient results.

**Table 2:** Recent evidence supports the use of nanohydroxyapatite in various aspects of dental care.

Type of Study	Purposes	Outcomes	Year	Ref.
In-vitro	Analyzed the activity of dental pulp cells on HANPs nanofibrous scaffolds for dentin regeneration.	PCL + 2%HANPs formulation showed 9 times higher mineralized matrix formation.	2022	^45^
In-vitro	Formulated resin-based composite with variable HANPs filler concentration, compared thermal properties.	Thermal stability is superior with Aluminium oxide-HANPs vs. Titanium dioxide–HANPs.	2022	^46^
In-vitro	Assessed odontogenic differentiation on HANPs-CMC scaffolds.	1:5 HANPs-CMC scaffolds evanesced cell viability, proliferation, and gene expression.	2022	^47^
In-silico	Evaluated molecular binding of PEEK-HANPs for orthodontic implants.	PEEK-HANPs showed a significant binding affinity with osteogenic markers, indicating the potential for osseointegration.	2022	^48^
In-vitro	Investigated GHMS scaffold for bone regeneration.	GHMS demonstrated superior bone regeneration compared to other materials.	2022	^49^
Animal Study	Compared HLA-HANPsNP combination for bone regeneration.	The HLA + HANP group showed superior bone formation, suggesting accelerated bone production.	2022	^50^
In-vitro	Developed and analyzed root repair material with HANPs.	HANPs-containing root repair material showed increased cell viability.	2022	^51^
In-vitro	Examined HANPs-coated implants for periodontal ligament cells.	Boron-doped HANPs improved the adhesion and proliferation of periodontal cells.	2022	^52^
In-vitro/ Animal	Studied HANPs composite for bone repair in diabetic defects.	HANPs composite showed evanesced bone repair and biocompatibility.	2022	^53^
In vitro	Investigated antibacterial activity of Fe/Cu-HAP coating on implants.	Fe/Cu-HAP coating displayed evanesced biocompatibility and antibacterial properties.	2022	^54^
In-vitro	Examined CIP-HANPs coated implants for antibacterial efficacy.	CIP-HANPs-coated implants showed sustained release and increased antibacterial activity.	2022	^55^
Clinical study	Explored HANPs effect on post-bleaching hypersensitivity.	Desensitizing agents did not affect bleaching outcomes but resulted in significant color change.	2022	^56^
Clinical study	HANPs were compared with fluoride to treat dentin hypersensitivity.	Fluoride and HANPs showed similar efficacy.	2022	^57^
In-vitro	Investigated remineralization potential of HANPs gel.	HANPs gel demonstrated superior surface remineralization compared to other agents.	2022	^58^
In-vitro	Examined HANPs resin infiltrant for enamel repair.	HANPs resin infiltrant showed improved resin penetration and mineral density.	2022	^59^
In-vitro	Compare various agents for preventing enamel lesions.	Fluoride varnish and mouthwash were effective in caries prevention.	2022	^60^
In-vitro	Investigated remineralization agents on enamel lesions.	CSP exhibited the highest microhardness, followed by HANPs and CPP-ACP.	2022	^61^
In-vitro	Assessed HANPs paste for remineralizing early enamel caries.	Homemade HANPs paste showed eHANPsnced remineralization potential.	2022	^62^
In-vitro	Tested antibiofilm activity of HANP suspensions.	Large nanoparticle HANPs exhibited eHANPsnced bacterial adhesion.	2022	^63^
In-vitro	Explored remineralization effects on primary teeth enamel.	Remineralizing agents equally increased microhardness and maintained enamel structure.	2022	^64^
In-vitro	Compared remineralization potential of various HANPs pastes with fluoride toothpaste.	Fluoride toothpaste and HANPs pastes showed improved enamel microhardness and fluorescence.	2022	^65^
In-vitro	Studied HANP effect on sealant properties.	HANPs sealant showed improved adaptation and fluoride release.	2021	^66^
In-vitro	Developed bilayer nanocomposite membrane for guided bone regeneration.	Membranes are suitable for periodontal applications, promoting bone regeneration.	2021	^67^
In-vitro	Fabricated bilayered tissue-guided membranes.	PA6/CSn-HA/PA6 scaffolds showed safety and osteo-conductivity for bone regeneration.	2021	^68^
RCT	Explored remineralization with HANPs dental products.	Dental lotion and toothpaste with HANPs-eHANPsnced remineralization.	2021	^69^
In-vitro	Tested HANPs adhesive on odontoblastic differentiation.	30% HANPs/SB promoted maximal reparative dentin formation, suggesting direct pulp capping potential.	2021	^70^

### Implantology

Coating titanium implants with a calcium phosphate-based substance has been demonstrated to eHANPsnce osteointegration and functional durability in implantology. An implant can be coated using a variety of procedures, such as dip coating, hydrothermal or sol-gel treatments, electrochemical or electrophoretic deposition, biomimetic, and ion beam-assisted deposition.^71^ Several aspects are considered while evaluating the effectiveness of coatings on dental implants. They include histomorphometrically analysis, cytotoxicity, removal torque, and biocompatibility. Studies in vivo are carried out to assess these elements and ascertain the efficacy of the coatings.^72^

Nano-hydroxyapatite (HANPs) is a popular material for coating titanium and stainless-steel implants due to its ability to eHANPsnce bone bonding and promote the formation of new bone tissue, resulting in improved bone-to-implant contact. The success of the implant and its interaction with the surrounding tissue is a testament to the effectiveness of these coatings. This success is determined by various factors, including the implant's chemical composition and surface roughness, as well as the size and shape of the HANPs coating. These aspects play a crucial role in the ability of osteoblasts to attach to the implant and promote bone formation.^73^ An additional advantage of using nano-hydroxyapatite (HANPs) as a coating material for dental implants is its unique ability to prevent the growth of Gram-positive and Gram-negative bacteria, ensuring the safety and efficacy of the implants.^71^^,^^74^

The presence of nano-HA coating on implants is advantageous as it helps to decrease inflammation since HA is a modulator for monocytes and macrophages, which are responsible for the initial inflammatory response.^75^ Grafting materials containing nano-HA, which have high porosity, are used as alloplastic materials, and they promote angiogenesis to eHANPsnce bone healing, allowing for early implant placement due to faster bone formation, with a healing period of approximately 4 months.^71^ The potential uses of nano-HA in treating bone defects caused by surgery or trauma are promising. While hydroxyapatite has been commonly used to treat such defects in the form of larger particles or blocks, its nanoscale particles have only recently been introduced as an injectable form in clinical practice, opening up new possibilities for bone defect treatment.^76^

When combined with stem cells or growth factors and placed on a scaffold, nano-HA is a practical material for tissue engineering and regeneration of bone or cementum, making it useful in oral surgical procedures such as cleft lip and palate repair and periodontal interventions.^77^ To create scaffold and bioceramic composites, it is possible to blend the bioceramic compound with the matrix or apply a ceramic coating onto the scaffold surface, resulting in a mineralized layer.^78^ These nano-HA-infused composite materials are helpful in dentistry treatment for conserving alveolar bone following tooth extraction, preventing bone loss, and facilitating successful implant implantation.^79^

### Dentin hypersensitivity

Dentin hypersensitivity occurs when exposed dentin causes pain in response to various stimuli, such as chemicals, temperature changes, touch, or osmotic pressure. This discomfort cannot be attributed to other dental issues or pathology.^80^ Nano-HA is commonly used to treat dentin hypersensitivity due to its bioactive properties. It facilitates the mineralization process and helps counteract the sensitivity caused by fluid movement within exposed dentinal tubules. This movement can excite pulp receptor cells and cause pain in response to various stimuli. By penetrating these tubules and acting as a mineralizing agent, nano-HA can block the tubule and halt the fluid flow, providing long-lasting relief from sensitivity and improving resistance to chemical and mechanical factors.^71^ Compared to other desensitizing agents, nano-HA is preferred for addressing hypersensitivity due to its ability to remineralize the tooth's surface by forming an appetite coating, which effectively counteracts the sensitivity.^81^

Limeback et al.'s study states that Dentin Hypersensitivity (DH) is a common issue that affects quality of life and often leads to invasive dental treatments. Hydroxyapatite (HA) has effectively reduced DH symptoms in clinical trials, showing superiority over fluoride and comparable efficacy to other desensitizing agents. This systematic review and meta-analysis, comprising 44 clinical trials, confirm the significant reduction in DH with HA-containing oral care products compared to placebo and fluoride, suggesting its potential as a preferred treatment option.^80^

### Bleaching

The teeth bleaching process involves releasing reactive oxygen species by the bleaching agents, which penetrate through the enamel and into the dentin to break down organic molecules. This transforms the compounds into lighter and more transparent shades.^82^ Bleaching gels with 30-35% hydrogen peroxide concentration are commonly used, but they can cause hypersensitivity in up to 70% of patients. To address this issue, the gel is often supplemented with rematerializing agents, including fluoride calcium and nano-sized hydroxyapatite, to minimize the risk of post-bleaching hypersensitivity.^83^ The enamel's microscopic surface defects and subsurface pores can lead to bleaching agent penetration and sensitivity. Still, nano-HA paste can effectively repair these defects and prevent the sensibility reaction.^84^

### Caries prevention

Nanotechnology is becoming more popular in the field of cardiology and prevention, as nano-HA is utilized to restore the demineralized dentin and enamel affected by caries; in the early stages of the condition, mineral ions are lost from the hard tissue due to acid attack from bacterial metabolism, while the collagen network is left intact.^71^ Nanoparticles such as nano-HA and bioactive glass are utilized to remineralize the organic scaffold affected by early-stage caries. These nanoparticles function as a substitute for the lost minerals or as a means of carrying the ions lost during the carious attack.^85^

Nano-HA is added to toothpaste for caries prevention to supply ions that reduce demineralization and eHANPsnce remineralization. The particles can penetrate tooth porosities and form a protective layer on the tooth surface.^86^ Acidic drinks can worsen erosion of the tooth's surface; hence, to reduce this effect, nano-HA has been added to sports drinks.^87^

## Importance of hydroxyapatite nanoparticles in dental implants

**Fig. 3:**
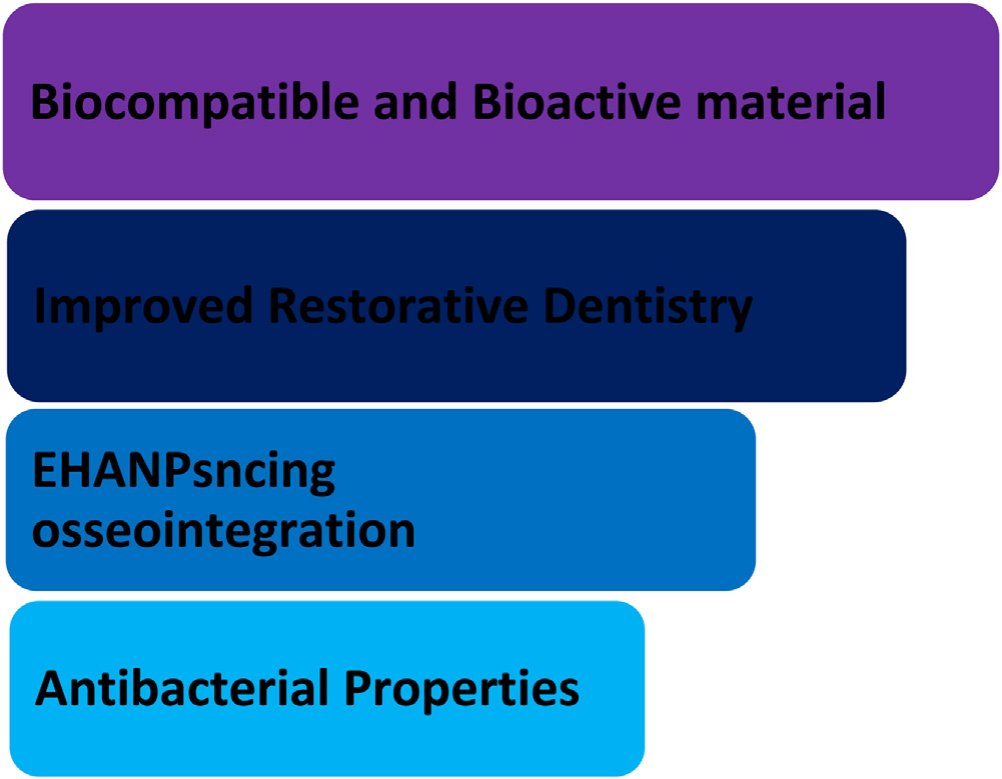
Importance of hydroxyapatite nanoparticles in dental implant.

Dental implants are the recommended treatment option for lost teeth when protecting surrounding tissues and ensuring adequate retention. Implants are typically used as screws, plates, staples, or root-formed implants. These implants are prepared from stainless steel and titanium alloys. Mainly titanium because of their adaptability and mechanical properties, but these materials have the disadvantage of not adjusting to the tissue environment locally. This issue is solved by hydroxyapatite (HAp).^88^ As a bioactive, osteoconductive, biocompatible, non-toxic, non-immunogenic, and non-inflammatory material, HAp is well regarded.^89^ These days, hydroxyapatite, whether synthetic or biologic, is used for bone regeneration and repair as scaffolds, blocks, and granules; it can also be used alone or in composites with polymers or other ceramics, or it can be coated on dental or orthopedic implants. The eHANPsnced integration of skeletal tissues to coated implant surfaces is necessary for the promising results of hydroxyapatite coatings.^90^ Hydroxyapatite nanoparticles are essential in dental implants. They are given in below Figure 3.

Because of their advanced osseous tissue integration, HAp coatings are increasingly crucial for dental materials, particularly implant surfaces.^88^ In general, calcium hydroxyapatite is referred to as HA. It is widely utilized in orthopedics for bone repair and implants, as a material substitution in dentistry, and as a filler in composites for medical applications because it is a significant component of bones and teeth.^91^ The impact of doped metal nanoparticles in HA on eHANPsncing the chemical and physical characteristics of dental implants is given below in Table 3.

**Table 3:** The impact of doped metal nanoparticles in HA on eHANPsncing the chemical and physical characteristics of dental implants.

Nanoparticles	Nanoparticles effect on dental implants	Ref.
Zinc Oxide Coating with Hydroxyapatite	Improve biocompatibility, mechanical properties, and osteoblast attachment.	^92^
Titanium Dioxide-coated HA	bone defects regenerating	^93^
Silver HA	EHANPsnced the biocompatibility and antimicrobial activity	^94^
HA nanocoating and titanium dioxide	Antibacterial activity and biological stability	^95^
Iron doping HA	Improving mechanical strength	^96^
Zinc and magnesium doped HA.	Improved mechanical characteristics	^97^

### Biocompatible and bioactive

The excellent biocompatibility of HAp-coated implants with epithelium, bone, and connective tissues has led to their widespread use in dental applications.^78^ The well-known biocompatible material, HAp, can be used for dental implants and orthopedic procedures instead of the vital mineral components of skeletal bones^98^ bioactive surfaces, including HA coatings, evanesce, bone adherence to orthopedic prostheses, and dental implants. Moreover, HA is used as a source material for bioactive coatings that are applied to orthopedic and dental implants, and it is also used as an abrasive to roughen the surfaces of metal implants.^92^ The mineral characteristics of natural bone are well-simulated by synthetic HA, which is recognized as an osteoconductive and biocompatible substance. EHANPsncing the biocompatibility of bone implants has been the goal of traditional micron-size HA for several years.^94^ Because hydroxyapatite (HA) shares many characteristics with the main mineral components of the human body's complex tissues, including bone and dental enamel, including bioactivity, biocompatibility, and low solubility in wet conditions, HA has found extensive use in biomedical and dental applications.^99^

### eHANPsncing osseointegration

One of the most crucial factors determining whether bone-anchored metallic implants in dentistry succeed or fail is osseointegration.^100^ HA is a well-known and commonly used substance frequently employed as relatively thick implant coverings. According to recently discovered procedures, implants can now be coated with a monolayer of HA nanoparticles. These thin HA coatings have been demonstrated to improve wettability, which raises the implant's surface energy. Elevating the surface energy is hypothesized to impact protein and cell adhesion, leading to eHANPsnced osseointegration.^101^ Implant-coating materials coated with HA nanoparticles have been reported to exhibit promising possibilities. They may improve osseointegration and biological fixation by forming a chemical link with the bone.^102^ Al_2_O_3_ nanoparticles are 1 of the top contenders for covering dental implants because of their high hardness, stability, and capacity to evanesce osseointegration.^103^ A nanoscale hydroxyapatite coating on an implant surface to investigate the acceleration and eHANPsncement of osseointegration compared to uncoated implants.^104^ Further evidence came from in vivo studies showing that Ti implant surfaces embellished with HA nanoparticles can promote osseointegration and the growth of new bone by improving osteocyte contact with the implant surfaces.^105^ A surface coated with nanoscale hydroxyapatite in the hopes that it may speed up and eHANPsnce osseointegration compared to uncoated implants.^106^

### Antibacterial properties

HAp is a biomaterial widely used in dentistry for metallic implant coatings or bone cavity fills. Its antibacterial properties have also led to its introduction in other fields.^107^ It has been demonstrated that the most potent antibacterial activity is provided by metal oxide nanoparticles of copper, silver, gold, or other non-metals such as hydroxyapatite, silica (SiO_2_), and chitosan through effective interactions with microbial membranes.^108^ In orthodontics, such nanoparticles can be used as a microimplant covering.^109^ When the diameters of the inhibition zones of 2 bacteria, such as Streptococcus mutans and Enterococcus faecalis, were measured and compared, it was found that the HA NPs' inhibition zone against the bacteria was more extensive than that of the chitosan. As shown in Table 4 below.

**Table 4:** The chitosan and hydroxyapatite nanoparticles' inhibitory zone diameters in millimeters against oral microbes.^97^

**Concentration** **(mg/mL)**	***Streptococcus mutans***		***Enterococcus faecalis***	
	Hydroxyapatite NPs	Chitosan NPs	Hydroxyapatite NPs	Chitosan NPs
*Inhibition zones in mm*				
2.5	12.08	11	9.3	8.6
5	14.43	13.20	11.3	10.3
10	18.3	16.2	14.5	12.4

In the dental environment, Hybrid Antimicrobial Nanoparticles (HANPs) may have their bactericidal properties degraded over time due to interactions with food particles, saliva, and microbial biofilm. Prolonged exposure is any amount of exposure that degrades or dilutes their antibacterial activity or causes the development of bacterial resistance. More studies are needed to understand how these characteristics oscillate over time, as there are few specific studies regarding the long-term efficacy of HANPs in dental applications.^110^ However, researchers are already finding solutions by adding some doping to solve the problems with saliva.

### Improved restorative dentistry

The qualities of materials now utilized in restorative dentistry can be improved by hydroxyapatite nanoparticles because of their high biocompatibility and bioactivity.^111^ It has also been examined whether using HA NPs as a component could eHANPsnce the qualities of current restorative dentistry materials.^112^ HAp can be used in restorative and preventive dentistry to remineralize early caries lesions on enamel, shielding teeth from dental degradation and caries.^113^ Because of its larger surface area compared to regular HA, nano-sized HA shows better sinterability and eHANPsnced densification, which could improve fracture toughness and other mechanical properties. When used to create restorative composites, nanoparticles demonstrated favorable mechanical and physical characteristics.^114^

## Synthesis of hydroxyapatite nanoparticles

Different methods, including dry techniques, wet chemical reactions, and mechanochemical reactions, can be employed to produce synthetic hydroxyapatite, with some of these methods capable of generating nanosized hydroxyapatite.^115^, ^116^, ^117^, ^118^ Challenges encountered during the synthesis of hydroxyapatite nanoparticles include the occurrence of phase impurities, aside from Calcium Phosphate salts, and the complexities in managing particle size, size distribution, morphology, crystallinity, and the extent of particle aggregation.^119^ Previous studies have investigated several synthetic procedures for producing hydroxyapatite nanoparticles, including hydrolysis, the sol-gel technique, mechanochemical methods, and wet-chemical precipitation.^120^ But they need to be more effective according to requirements. For instance- HA NPs obtained via hydrolysis, a low-temperature process, exhibit grain sizes ranging from 20 to 50 nm and display diverse morphology while maintaining a high phase purity of HA NPs.^121^ Alternatively, the 'sol-gel' method produces HAnps with crystalline sizes between 20 and 60nm, also with diverse morphology and high phase purity.^122^ The ideal circumstances for controlling the synthesis of nanoparticles for dental implant use depend on the parameters for the synthesis methods, such as sol-gel and hydrothermal processes. The right size, shape, and purity of nanoparticles are produced by managing these variables, which are essential for enhancing treatment outcomes and reducing side effects. Like the sol-gel technique, stable colloidal dispersion at the specified solvents is required for the selected medium. To determine if the nanoparticles satisfy the necessary conditions for usage in dental applications, they should be described using standard techniques.^123^

**Fig. 4:**
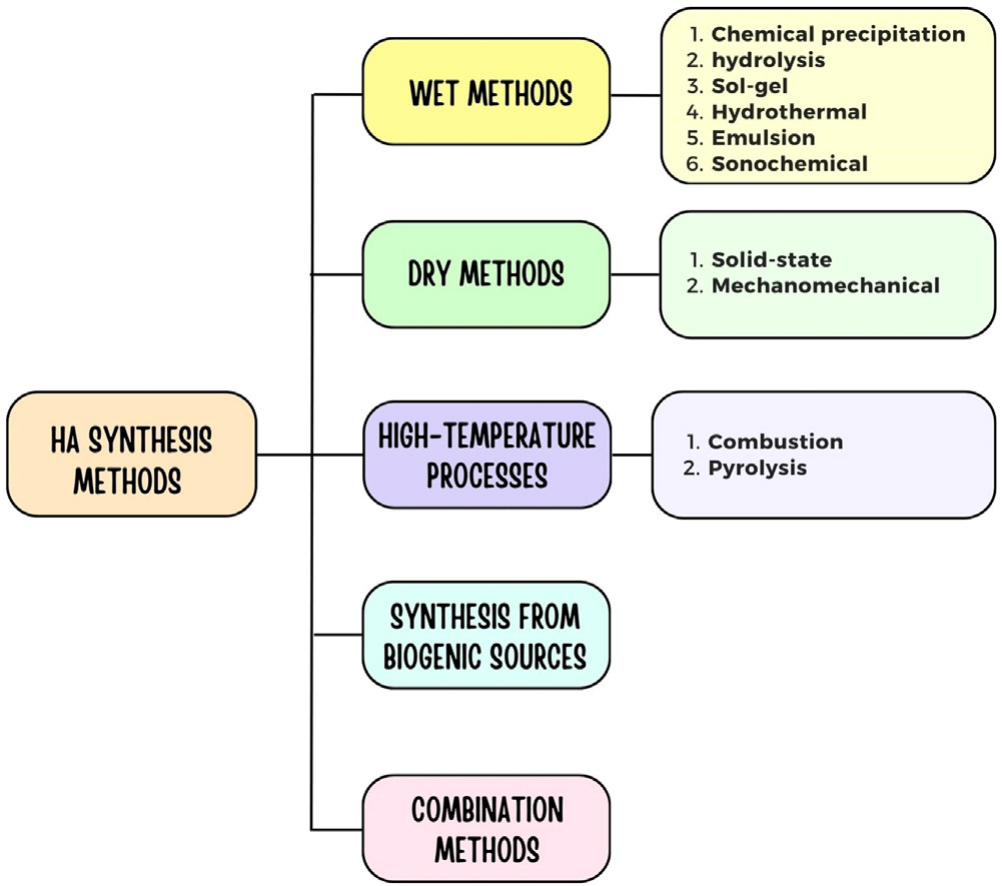
HA synthesis methods.

Another approach involves mechanochemical procedures, enabling the synthesis of nanosized hydroxyapatite with an average particle size of 25nm. However, this method yields non-stoichiometric HA NPs with low phase purity despite its cost-effectiveness and operability at room temperature.^124^ Several methods have been outlined in the literature to achieve precise control over the structure of synthetic HA. Szcześ et al.^125^ categorized the preparation techniques into 5 groups: (1) wet methods, including chemical precipitation, hydrolysis, sol-gel, hydrothermal, emulsion, or sonochemical processes; (2) dry methods, encompassing solid-state and mechanomechanical approaches; (3) high-temperature processes, like combustion and pyrolysis; (4) synthesis utilizing biogenic sources; and (5) combination methods, such as hydrothermal-microemulsion, hydrothermal-mechanochemical, hydrothermal-hydrolysis, and others (Figure 4).

Conventional methods for producing hydroxyapatite nanoparticles (HA NPs) often involve prolonged processing durations, sometimes up to 24 hours, and harsh processing conditions such as high pH, temperature, or ultrasonication. These conditions can lead to HA NPs with properties diverging from those of biological apatite. However, employing Simulated Body Fluid (SBF) with a salt composition akin to human blood plasma and heightened precursor concentrations has proven effective in circumventing these challenges. By adopting this approach, “bone-like” carbonated HA NPs can be prepared in just 3 hours, resulting in HA NPs crystals characterized by a uniform rod shape and dimensions measuring approximately 27 nm × 7 nm. The abbreviated processing time associated with this technique holds significant promise, particularly for coating metallic biomaterials with HA NPs, as it can potentially eHANPsnce productivity.^126^, ^127^

Table 5 outlines several techniques for synthesizing hydroxyapatite, such as CP, Sol-gel (SG), and hydrothermal methods. Each method yields distinct arrangements and chemical compositions. However, attaining high aspect ratios, precise stoichiometry, and high crystallinity presents challenges across these techniques.

**Table 5:** Hydroxyapatite nanoparticles are made using diverse methods, each with unique features.

Synthesis Method	Synthesis Duration	Particle Size	Morphology	Temp. (^o^C)	Structural Integrity	Cost	Phase Purity	Size Distribution	Ref.
Sol-gel	-	> 0.001 µm	Diverse	37-85	Variable	Variable	Variable	Narrow	^128^
Solid-state	-	> 2 µm	Diverse	1150 ± 100	High	Low	Low	Wide	^129^
Flux cooling	< 24	18×2.1 nm	Hexagonal prism	200	High	Variable	Variable	Wide	^130^
Microemulsion	> 24	> 1	Needle-like	RT	Low	High	Variable	Narrow	^131^
Electrospinning	> 24	10×30 nm	Fibrous	-	High	Variable	Variable	Variable	^132^
Core-shell	>24	> 0.01 µm	Diverse	60-120	Variable	Variable	Variable	Narrow	^133^
Microwave irradiation	< 24	100×25 nm	Diverse	-	High	Variable	High	Narrow	^134^
Hydrothermal	< 24	> 0.05	Needle-like	150-400	High	High	High	Wide	^135^
Electro spraying	-	75×40 nm	Diverse	-	Variable	Low	Variable	Wide	^136^
Chemical precipitation	> 24	> 0.1 µm	Diverse	RT	Low	Low	Variable	Variable	^137^
Self-propagating combustion	< 24	> 0.45 µm	Diverse	170-500	Variable	Low	High	Wide	^138^

**Fig. 5:**
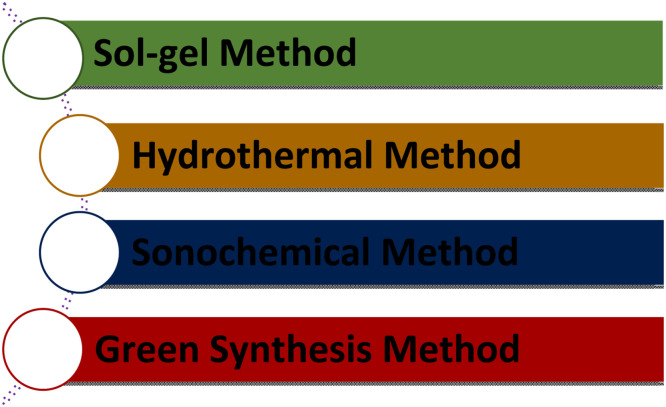
Different types of hydroxyapatite nanoparticle synthesis processes.

Despite using various chemicals and reactants, significant processes produce HA powders.^139^ It has been used in several biological applications, including dental and bone fillers. Several methods based on wet chemical processes have been developed during the past few decades to precisely regulate the size, shape, and content of HAp nanoparticles.^140^ Some of them are shown below in Figure 5.

A sol, or a colloidal solution of solid particles, is changed into a 3-dimensional solid phase network during the sol-gel processes. To eliminate organic residues, this procedure involves mixing precursors (usually alkoxides) and aging, gelating, drying, and calcining them.^141^ One way to conceptualize hydrothermal synthesis is as a higher-temperature chemical precipitation. Organic modifiers are frequently used to regulate the morphology and structure of the crystals produced using this approach.^142^, ^143^ Using sonochemical techniques makes homogeneous particle production possible, and the chemical reaction in the presence of ultrasonic radiation invariably produces nanosized products.^144^ The production of HA in the emulsion prevents agglomerates from forming and gives control over the size and shape of the resulting particles. Water-in-oil, oil-in-water, and water-in-oil-in-water double emulsions can all be created utilizing emulsion synthesis and different types of surfactants.^145^ More on various types of synthesis processes of hydroxyapatite nanoparticles are described below in Table 6.

**Table 6:** Different hydroxyapatite synthesis techniques.

Methods	Advantages	Disadvantages	Ref.
Sol-Gel	The resulting HAp particles will have superior surface area, excellent transparency, and outstanding dimension structure.	There is frequent volume shrinkage and cracking during the drying process.	^146^
Hydrothermal	The hydrothermal treatment improves the morphological and mechanical characteristics of HAp nanoparticles.	Utilizes temperatures above ambient and high pressures.	^147^
Sonochemical	The sonochemical approach improves energy economy and reaction speed.	The ultrasonic frequency affects the sonochemical reduction rate.	^148^
Ultrasound–Microwave	Particle accretion is reduced by ultrasound, and processing time is shortened by microwave irradiation.	Nanoparticles exposed to uniform microwave irradiation become less crystalline.	^149^

### Hydrothermal method

Several documented methods for synthesizing HAp nanoparticles include a hydrothermal method.^150^ Hydrothermal techniques are typically utilized to synthesize nanofibers or nanowires.^151^ On the other hand, as the hydrothermal pressure or temperature increases during the hydrothermal treatment, the precipitates' Ca/P ratio improves. For that reason, HAp nanoparticles can be prepared using this method.^152^, ^153^ The hydrothermal method appears to offer the most benefits of all the hydroxyapatite nanoparticle synthesis methods.^154^ However, A key disadvantage of hydrothermal treatment is that it can be challenging to regulate the shape and dispersion of HAp particles.^155^ A progression of the hydrothermal method is Microwave Hydrothermal Synthesis (MHS).^156^ MHS is a microwave-assisted hydrolysis technique that uses a closed reaction vessel and a temperature more remarkable than the boiling point of water to cause a chemical reaction or breakdown between precursors. The early-stage synthesis of the HAp method was successfully achieved by developing an easy-to-use, economical, and ecologically favorable technology.^157^, ^158^

Relevant research examples of the preparation of hydroxyapatite nanoparticles by hydrothermal method. The calcium and phosphorous sources were calcium nitrate [Ca (NO_3_)_2_] and disodium hydrogen phosphate (Na_2_HPO_4_), respectively, and the pH was adjusted with ammonium hydroxide (NH_4_OH, 29%). Every reagent that is utilized is analytical grade. After stirring the 0.15 M (Na_2_HPO_4_) solution, 0.25 M Ca(NO_3_)_2_ was added dropwise until the Ca/P molar increased to 1.67. Next, STPP and Na_5_P_3_O_10_ were added to the mixture. To create a precursor of HAp, the pH level and temperature were changed to 8-10 and 40-60°C, respectively. After being moved into a stirring hydrothermal reactor, the residue was left to sit at 160°C for 4-12 hours. Following cooling, the HAp precipitate was cleaned and filtered using ethanol and deionized water in that order. Finally, the residues were dried for 24 hours at 50°C in a laboratory oven.^159^

### Sol-gel method

Compared with conventional procedures, the sol-gel method may significantly improve the outcomes of the chemical homogeneity HAp and has certain advantages at the molecular level of the phosphorus precursors and calcium mixing.^160^ The well-known intrinsic benefits of the sol-gel process, which include uniform molecular mixing, low processing temperatures, and the capacity to produce thin films, bulk amorphous monolithic solids, and nanocrystalline powders, have made the approach popular.^161^, ^162^ When HAp is prepared via the sol-gel process, a fine-grain microstructure with a mixture of crystalline-structured nano-to-submicron particles is often formed. The contact and stability at the artificial/natural bone interface seen in vitro and in vivo settings have been claimed to be much improved by these crystals.^163^ The calcining and sintering temperatures are lowered due to the sol-gel powders' high reactivity.^164^

Relevant research examples of the preparation of hydroxyapatite nanoparticles by sol-gel method. The PCC and (NH_4_)_2_HPO_4_ of the egg shells were combined by adjusting the aging period (24, 48, 72 hours), pH (9, 10, 11), and the ratio of Ca to P (1.57; 1.67 and 1.77). While (NH_4_)_2_HPO_4_ was dissolved with aqua dust, PCC was dissolved in 0.3M HNO_3_. A glass beaker was also used to combine the PCC and (NH_4_)_2_HPO_4_ solutions. Until the gel formed, the solution was agitated for 3 hours at 300 rpm. After that, the same procedure was carried out using the modified variable. After the aging procedure, the gel is dried for 24 hours at 80°C in an oven. After carefully washing the resulting particles in distilled water to remove the hydroxyapatite from the remaining reactants, they were allowed to dry in the oven for 3 hours. Lastly, the solids were calcined at 500°C for 1 hour.^165^, ^166^

Doping with hydroxyapatite can develop tantalum pentoxide (Ta_2_O_5_) and Strontium Ion. The additive gel makes a strong bond between the coating and substrate.^167^ Polyethylene Glycol can be used as a gel to keep a strong HAp-like structure, like natural bone.^168^ Optically transparent and crack-free, this firm can be formulated with almost 1600 Å thickness. So, this sol-gel method can effectively generate a thin film.^169^ Again, layer-by-layer, a thin surface can be generated with this method, and the film can be controlled at the Angstrom level. The presence of dielectric properties can make the movie denser and more classified.^170^

### Green synthesis method

**Fig. 6:**
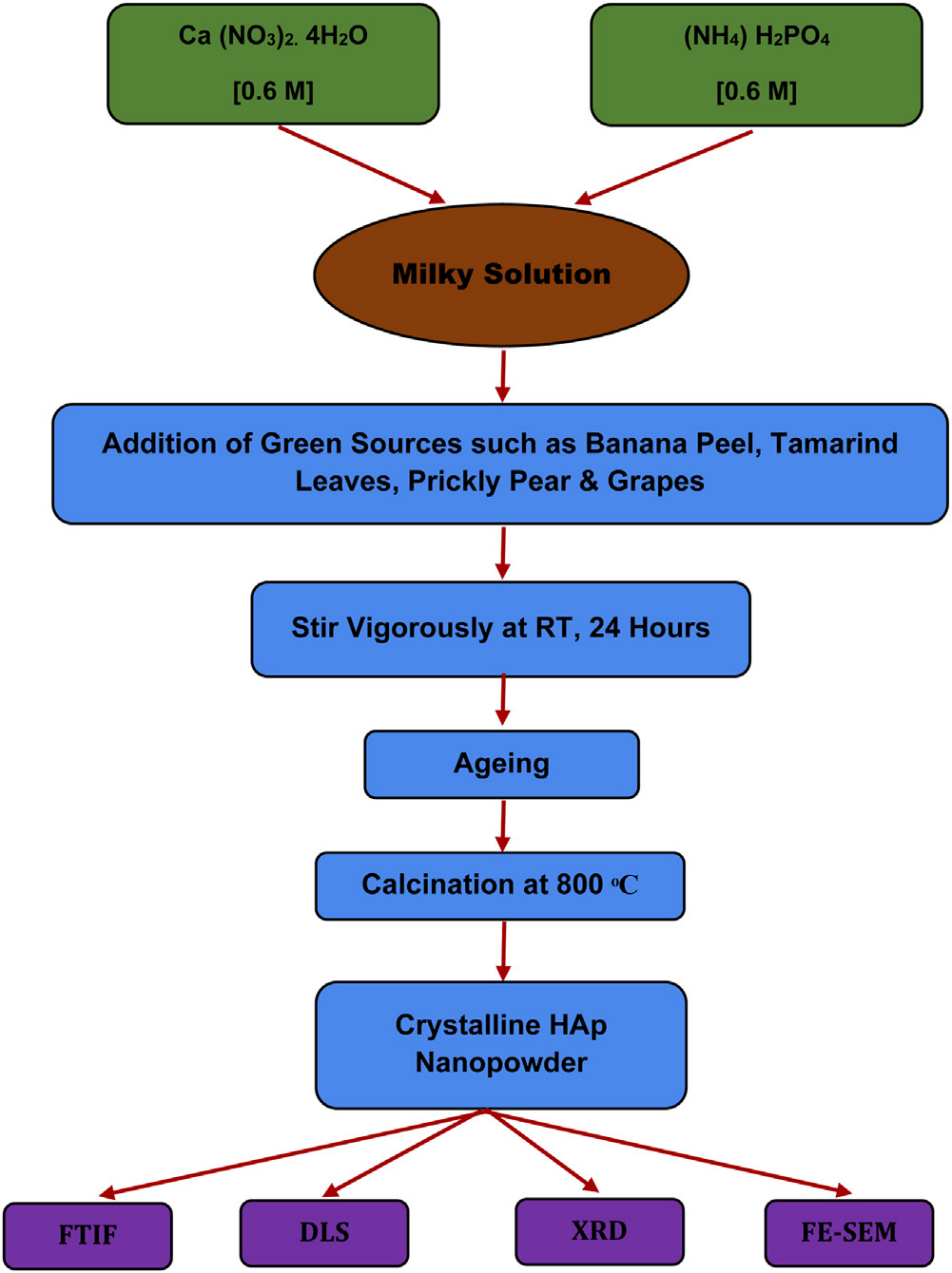
HAp nanoparticle production using a green synthesis technique.

An alternative to the current standard synthesis methods is the green synthesis method. This promising approach uses less energy and chemicals and substitutes naturally occurring materials for precursors, thereby reducing the cost of adsorption.^171^ Due to its accessibility, biocompatibility, and lack of need for hazardous or unnecessary chemicals, green synthesis of nanoparticles has drawn increased interest.^172^ It is uncommon to find reports of green synthesis of HAp nanoparticles utilizing plant leaf extract. Consequently, from the perspective of materials processing, it is crucial to synthesize HAp nanoparticles with antibacterial capabilities employing various plant extracts.^173^ The utilization of green leaf extract in the green synthesis of HAp has not been extensively studied. Therefore, adding leaf extracts to HAp during production is crucial to boosting its antibacterial qualities.^174^ It was found that HAp, which was created utilizing green synthesis techniques and a bio template made of plant extracts, improved the material's morphological and crystallinity qualities.^175^ Because it is safe, biocompatible, and requires no hazardous or unnecessary ingredients, synthesizing nanoparticles using biological or green chemistry has drawn far more interest than physical or chemical methods. Green chemical substances are used instead of hazardous materials due to concerns about human health and the environment.^176^ Relevant research examples of the green synthesis techniques^177^ are given below in Figure 6.

The green synthesis technique is a cost-effective process due to the availability of ingredients in the environment. The most substantial phenomenon is that bio-waste, i.e., eggshells, shows high potential in mechanical and biological activities in medical treatment. Due to the presence of calcium and ammonium dihydrogen phosphate, the ultrasonication method is perfectly demonstrated in biomedical engineering to ensure the desired powder shape.^178^ The possibility of green synthesis approaches for hybrid organic-inorganic nanocomposite materials (HANPs) is discussed. However, there is little information on how these procedures may be scaled up for practical use.^179^ It is essential to their successful development to examine how these sustainable methods might be modified for use in larger-scale production processes without sacrificing the HANP's inherent qualities and therapeutic effectiveness.^180^ Green synthesis of hydroxyapatite (HA) nanoparticles has several benefits over conventional methods, including:

• **Environmental sustainability**

Green synthesis uses nontoxic, biodegradable, and renewable materials and is less polluting than traditional methods.^181^ In pharmaceutical technology, drugs can be made less poisonous and harmful by ensuring green chemistry, which leads to green synthesis.^182^ In biotechnology, it helps to keep ecological balance by providing sustainable environmental matrices.^183^

• **Cost-effectiveness**

Green synthesis can save up to 40% on costs compared to conventional methods.^184^ Plant extracts and other biological mechanisms produce significant possibilities for the healthcare sector to produce biocompatible and cost-effective particles.^185^ Microalgal biomass compounds can also be part of this, which is not limited to resources and is also cost-effective^186^ the dental industry benefits from this synthesis method for HAp nanoparticles.

• **Improved properties**

Green synthesis can produce nanoparticles with enhanced biocompatibility, thermal and electrical conductivity, and catalytic activity.^187^ It enriches mechanical, chemical, magnetic, electronic, and optical properties, which are higher than conventional methods.^188^ HAp needs all these characteristics for dental implants, which can be obtained only using green synthesis.

• **Controlled shape and size**

Green synthesis can produce nanoparticles with controlled shape and size. The size and structure of NPs are influenced by the type of biological entities present in varying quantities and reducing organic agents.^189^ In the medical industry, the variation of sizes depends on the synthesis of nanocomposites for multipurpose usage. HAp can be fabricated in microstructural, nanostructural, and other ways to control desired applications in biomedical sectors.^190^

• **Simple and efficient**

Green synthesis is a simple and efficient method for producing nanoparticles. Where every other technique is toxic and costly, the green synthesis method is considered better in advanced healthcare.^191^ As it is extracted from plant and living properties, it has a more splendid view of future resource availability. It has good ecological and cost-effective records for using biomedical technology.^192^

To increase the process's acceptability, methods like process optimization, scaling up, or incorporating green chemistry principles can make synthesis processes like sol-gel and hydrothermal more economically viable for medical applications without sacrificing effectiveness.^193^ Optimizing these material synthesis processes to reduce energy, reaction time, and material waste has the potential to significantly reduce costs. Other raw materials or mechanosynthesis techniques may result in more economical manufacture without compromising the performance qualities required for medical applications.^194^, ^195^

However, there is still a requirement to set the perfect method for synthesis according to patient needs. Multiple patients have different complexities, and the synthesis process is varied. Several reasons are responsible for dental failure, which leads to implant teeth. Some of these are due to biological diversions, such as males being more likely to suffer than females after a certain age. Some hereditary diseases like hypertension, coronary artery disease, and pulmonary disease lead to dental damage. The after-effects of some diseases, such as steroid therapy and chemotherapy, also cause dental problems.^196^ Variations in the technique are essential for synthesizing HAp nanoparticles due to multiple health issues. Some patients are about to lose their teeth due to bacterial infections. Hydrothermal process is an excellent choice as it ensures antimicrobial properties.^197^ Another reason for the implant is that gum recession HAp helps with dentin hypersensitivity. The sonochemical process resolves the dentine problem by coating.^198^ Some patients are facing dental problems because of nerve and tissue damage. The hydrothermal technique fabricates the required particles in damaged tissue, repairing teeth.^199^ Gum disease and tooth loss are fully responsible for Rheumatoid Arthritis. For this reason, a pure form of HAp extraction is required, which can be obtained from the microwave-assisted combustion method.^200^ Again, periodontal disease leads to tooth loss, which is affected due to diabetes problems. A patient who has diabetes has infections between the bone of joining teeth and gum tissue. In this case, the sol-gel method is the only solution for mitigating this gap and providing a link between teeth and bone.^201^

## Characterization processes of hydroxyapatite nanoparticles

Many attempts to construct nanocomposite scaffolds with superior mechanical, biocompatibility, bioactivity, and biodegradability have been made in the last few decades by combining bioactive materials with biocompatible and biodegradable polymers.^202^ Sales of biocompatible substances able to replace, substitute, or fill in bone or teeth are predicted to reach several billion euros worldwide. This global quest is currently expanding significantly. One of the bioceramic materials with the potential for replacing teeth and bone artificially is calcium phosphates, specifically HAp (Ca_10_(PO_4_)_6_(OH)_2_).^203^ In the last ten years, magnetite/HA NPs have attracted plenty of attention because of their potential use in therapy and regulated drug administration.^204^ HAp is a bioceramic substance utilized to minimize the operatory time for prosthetic encompassing, protein cleansing, bone trauma mending, and additional consolidation and substitution in bone remedies because it stimulates bone neoformation when in contact with physiological tissue.^205^

Figure 7, Fig. 8.

**Figure 7:**
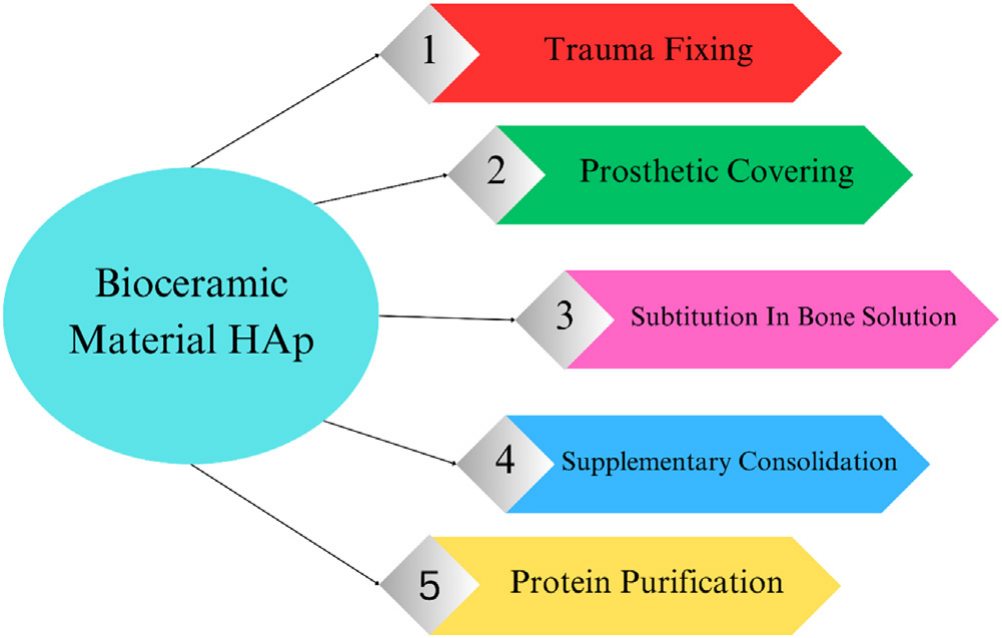
Significance of HAp as a bioceramic material.

**Fig. 8:**
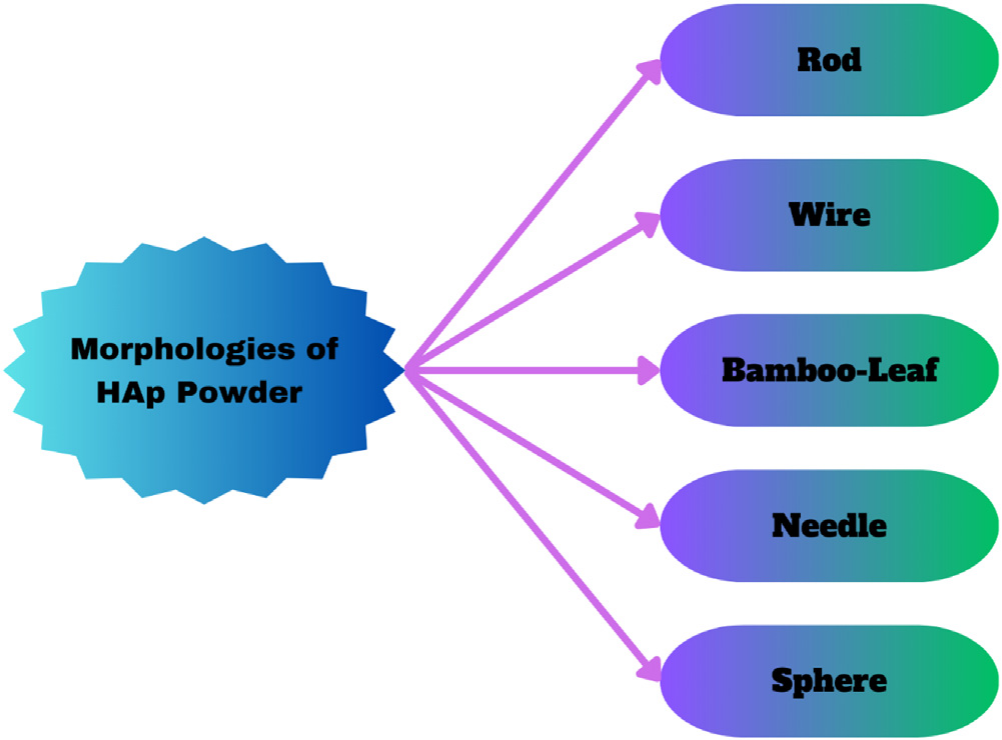
Morphologies of HAp powders.

HAp, Ca_10_(PO_4_)_6_(OH)_2_, is the most critical non-organic ingredient found in human solid tissues, including bones, dentine, and enamel.^206^ HAp is a bioceramic in biomedical applications due to its exceptional biocompatibility and excellent inductivity for skeleton ingrowth.^207^, ^208^, ^209^, ^210^ The primary inorganic component of natural bone is [HAp, Ca_10_(PO_4_)_6_(OH)_2_], which is widely utilized in a variety of biomedical applications.^211^

Thermodynamically speaking, HAp is the longest-lasting crystalline phase of the Ca_3_(PO_4_)_2_ ions in biological fluid. The primary mineral component of teeth and other calcified tissues, synthesized HAp, has exceptional bioactive properties such as osteoconductive and cytocompatibility because of its maximal correspondence to the mineral portion of bone tissue.^212^, ^213^, ^214^ One type of Ca_3_(PO_4_)_2_ that resembles human rigid tissues is called HAp. Many methods can be used to make it chemically; the 1 proposed in this work is based on moist precipitation via chemicals. It is the main element of enamel and bone.^215^ As the main non-organic complex component that makes up the muscular tissues in bones, HAp has good biocompatibility, bioactivity, no immunogenicity, and osteoconductive qualities. It can also be utilized as a crucial implant ingredient.^216^ Synthesized HAp is the most promising since it closely resembles the chemical structure and makeup of the mineral phase of bone.^217^, ^218^, ^219^ HAp nanoparticles were created using the wet chemical precipitation process at room pressure. HAp powders with various morphologies, including sphere, rod, needle, wire, and bamboo-leaf-like shapes, were produced by adjusting the synthesis conditions. Particle size and dispersibility were found to be impacted by drying techniques, dispersant species, and solvent systems.^220^

**Fig. 9:**
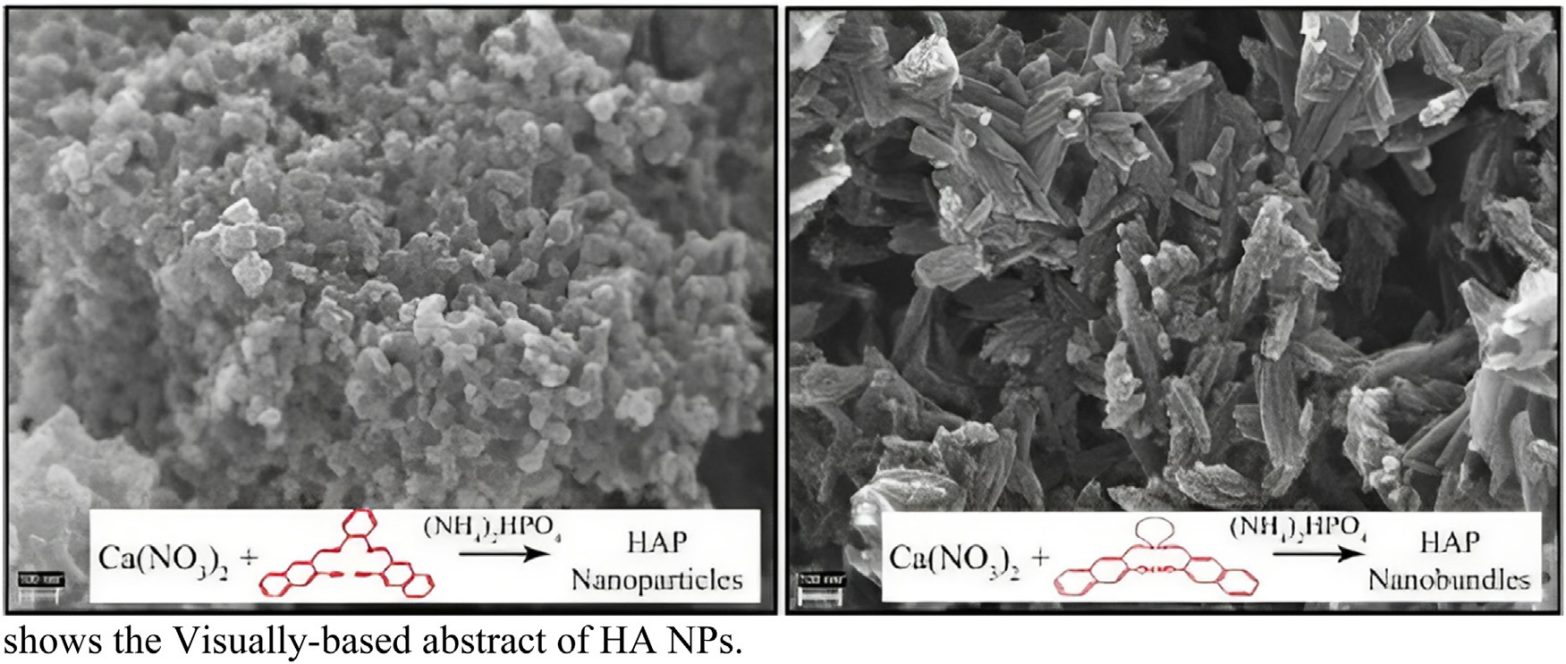
HAp nanocrystals.^226^

Studies on characterization and optimization were done using the direct precipitation method to produce HAp nanoparticles.^221^ It also shares a remarkable crystallographic and chemical structure with bone minerals, typically containing 25-75% HAp through mass and a calcium-to-phosphate balanced proportion of 1.67.^222^, ^223^ HAp nanocrystallites were created using the (M-H) technique at temperatures between 100 and 140°C using calcium CaH_8_N_2_O_10_, H_3_PO_4_, and C_2_H_8_N_2_ as material sources. The resulting reaction products were investigated using FTIR, SEM, and XRD.^224^ The hydrothermal synthesis process was used to create HAp NPs, which were then wholly characterized using techniques such as TEM, RS, FTIR, and XRD.^225^ Several methods, including TEM, FE-SEM, and XRD, were employed to characterize the HAp nanocrystals. Based on the FE-SEM and TEM micrographs, it was found that the ligands' coordination mode had an impact on the HAp powders' shape and crystallinity.^226^ Figure 9 shows the Visually-based abstract of HA NPs.

Table 6 provides information on the benefits and challenges of spectroscopic and direct visualization techniques that characterize HA in various forms, such as nanoparticles, rods, discs, and powder. These techniques are utilized for characterization purposes.^227^ Fourier Transform Infrared (FTIR), Raman spectroscopies, and X-ray techniques have been commonly employed to characterize HA. FTIR and Raman spectroscopic techniques are used to verify the chemical composition of HA by exploiting the material's specific characteristics for identification purposes.^228^, ^229^ X-ray Photoelectron Spectroscopy (XPS) is a spectroscopic technique that offers surface-sensitive analysis and quantification of elemental composition with high precision, down to the parts per thousand range. It also provides valuable insights into the empirical formula, chemical state, and electronic state of the material's constituent elements.^230^ Energy Dispersive Spectroscopy (EDS) is a crucial analytical approach to Scanning Electron Microscopy (SEM). Morphological details such as size, shape, and dispersion of HA nanoparticles can be obtained through direct visualization techniques such as SEM, Transmission Electron Microscopy (TEM), Confocal Laser Scanning Microscopy (CLSM), and Atomic Force Microscopy (AFM).^231^ In some cases, SEM may not provide conclusive information regarding HA nanoparticle dispersion, shape, and size. Therefore, complementary techniques like TEM and AFM are necessary for a more comprehensive analysis Table 7.^227^

**Table 7:** Classification, Benefits, and Challenges of various methods used to characterize HA nanoparticles and inorganic powder materials (Raman spectroscopy rather than FTIR typically analyzes these).

Type	Method	Benefits	Challenges	Ref.
**Spectroscopic**	Fourier Transform Infrared Spectroscopy (FTIR)	This method measures the intensity of a specific wavelength range at a time, requires no external calibration, and is precise in its results. It can accurately detect even small quantities of impurities or contaminants.	FTIR spectroscopy is not a simple technique for analyzing inorganic materials.	^232^
**Spectroscopic**	Raman spectroscopy	Raman spectroscopy provides precise chemical identification of materials, and inorganic materials can be analyzed more easily with Raman than with FTIR spectroscopy.	Due to the weak Raman effect, detection requires a susceptible and optimized instrument. Additionally, fluorescence from the sample or impurities can obscure the Raman spectrum, and the sample may be destroyed, or the Raman spectrum may be hidden due to the intense laser radiation used for heating the sample.	^233^
**Spectroscopic**	X-ray photoelectron spectroscopy (XPS)	This method offers distinct details about the chemical composition of a material.	This method has slow processing time, inadequate spatial resolution, and necessitates a high vacuum environment.	^234^
**Spectroscopic**	X-ray Diffraction Spectroscopy (XRD)	This technique is fast and rapid, with a processing time of under 20 minutes. It delivers an unambiguous mineral identification, and the data interpretation is relatively straightforward.	Homogeneous and single-phase materials are preferable for identifying an unknown substance. However, peak overlay may affect identification accuracy, particularly for high-angle reflections.	^235^
**Spectroscopic**	Energy Dispersive X-ray Spectroscopy (EDS)	The chemical microanalytical technique is combined with SEM to provide unique peaks that reflect the atomic structure of the atoms. It is a fast and flexible technique.	This method is relatively less precise.	^236^
**Direct Visualization**	Scanning Electron Microscopy (SEM)	This method allows for direct visualization and provides high resolution.	The preparation of samples may result in the aggregation of nanoparticles.	^236^
**Direct Visualization**	Transmission Electron Microscopy (TEM)	This method allows for direct visualization and provides high resolution.	Challenges in the sample preparation process can lead to nanoparticle aggregation, and there is a risk of electron beam damage. Additionally, this technique has a bias towards atomic species that are electron-dense.	^237^
**Direct Visualization**	Atomic Force Microscopy (AFM)	This method provides a high resolution and can produce a 3-dimensional profile.	This method has a slow scanning speed and a limited scanning area.	^238^

**Fig. 10:**
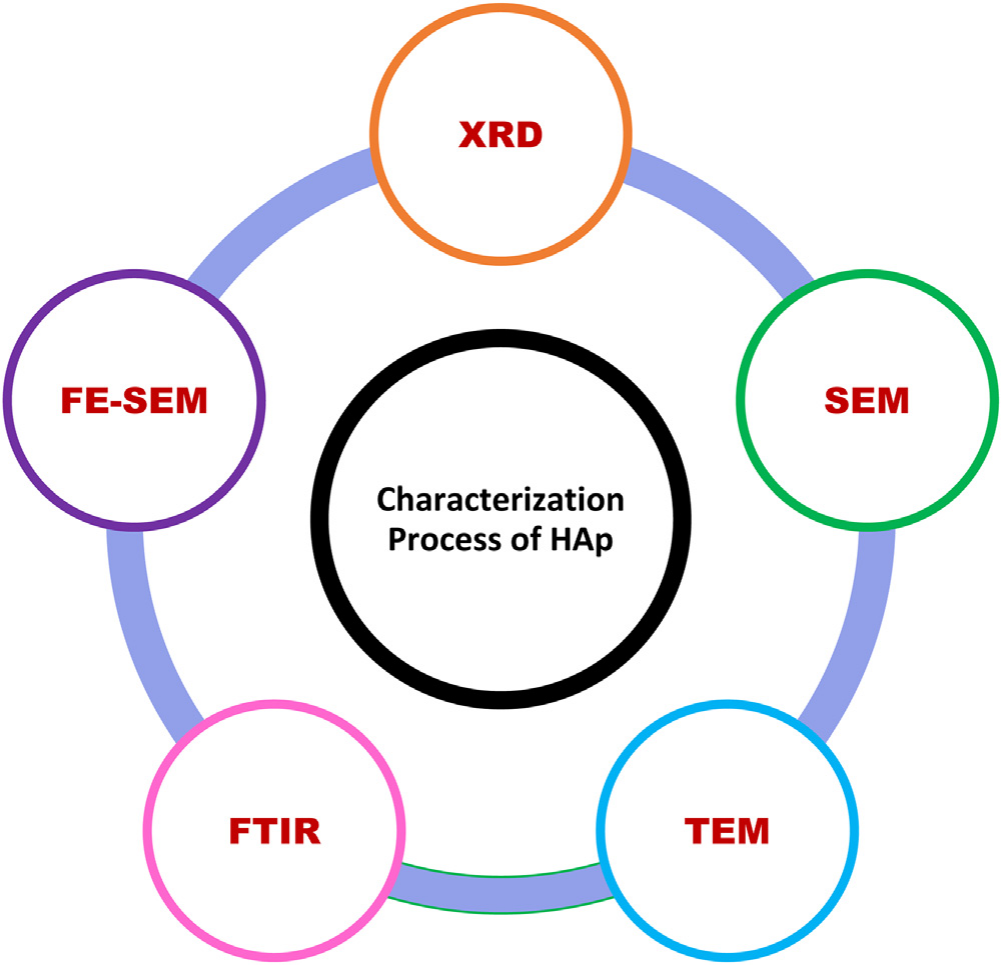
Graphical view of characterization processes of hydroxyapatite nanoparticles (HAp).

Figure 10 simulates the most obvious characterization processes of Nano-Hydroxyapatite, which are used for specific phenomena.

### X-ray diffraction (XRD)

**Fig. 11:**
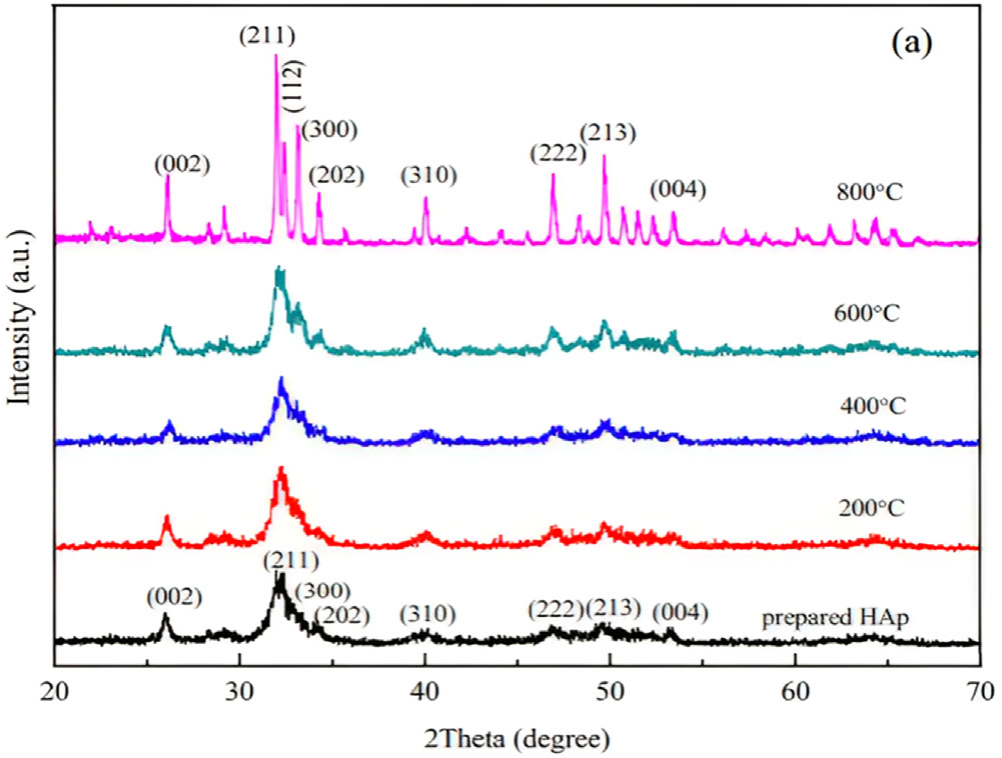
Prepared HANPs and HANPs sintered at 200, 400, 600, and 800 degrees Celsius: XRD patterns.^244^

XRD analysis determines the nanoparticles' stage identity and crystalline form. The equation of Debye-Scherrer, D=Kλ/β cos θ (3.1), was used to calculate the crystalline size of the particle. Here, λ stands for the XRD, the length of the wave, β for the entire 1/2 highest, and θ for Bragg's angle.^239^ The XRD patterns show that within 5 minutes of sonication, HAp production began.^240^ XRD stands for X-ray diffraction, and AFM stands for atomic force microscopy. The resulting dual-ion-doped HA NPs have a stable HAp structure devoid of impurity peaks.^241^ It is impossible to determine the volume of the nanoparticles extracted from patterns created by XRD since a particle contains several nanoscale or microscale crystals. The computation of size is related to the crystals rather than the particles since an X-ray may look past the size of the crystal to disclose data.^242^ XRD analysis was used to identify the virgin HAp and HAp collection stage contaminants with different doping percentages.^243^ Figure 11 illustrates the XRD pattern of HAp [Ca_10_(PO_4_)_6_(OH)_2_] nanomaterials.^244^

**Fig. 12:**
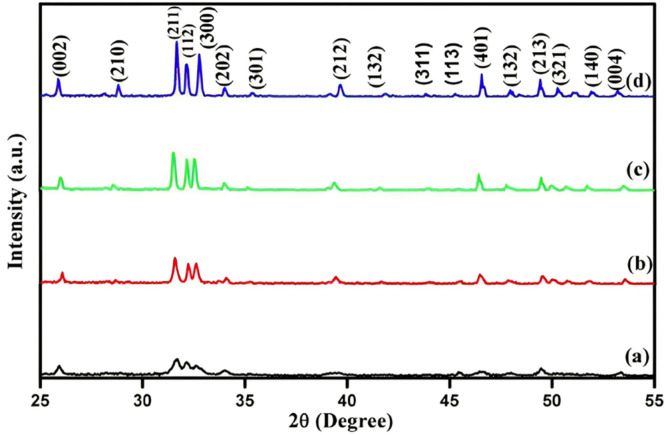
XRD patterns of HAp at various annealing temperatures (a) as prepared, (b) 700°C, (c) 800°C, and (d) 900°C are shown.^238^

Figure 12 shows the XRD structures of the HAp annealing at different temperatures, including 700, 800, and 900°C when ready. The peaks of all 4 diffraction patterns were assessed against the standard HAp provided by the (ICDD card # 09-432).^238^

An X-ray Diffractometer (X'Pert Pro MPD Diffractometer, Panalytical, Alnelo, Netherlands) with a scanning range of 0°-60° was used to determine the XRD pattern of the generated nano HAp powder and electrospun nanofiber mats to evaluate various crystalline phases and structures.^245^

#### Field emission scanning electron microscopy (FE-SEM)

An inorganic substance used in dental and bone tissue engineering is HAp. Using an SEM and FE-SEM, the morphological characteristics of the samples were examined.^246^ Researchers have used FE-SEM in several studies to examine the morphological characteristics of HA NPs. For example-

**Fig. 13:**
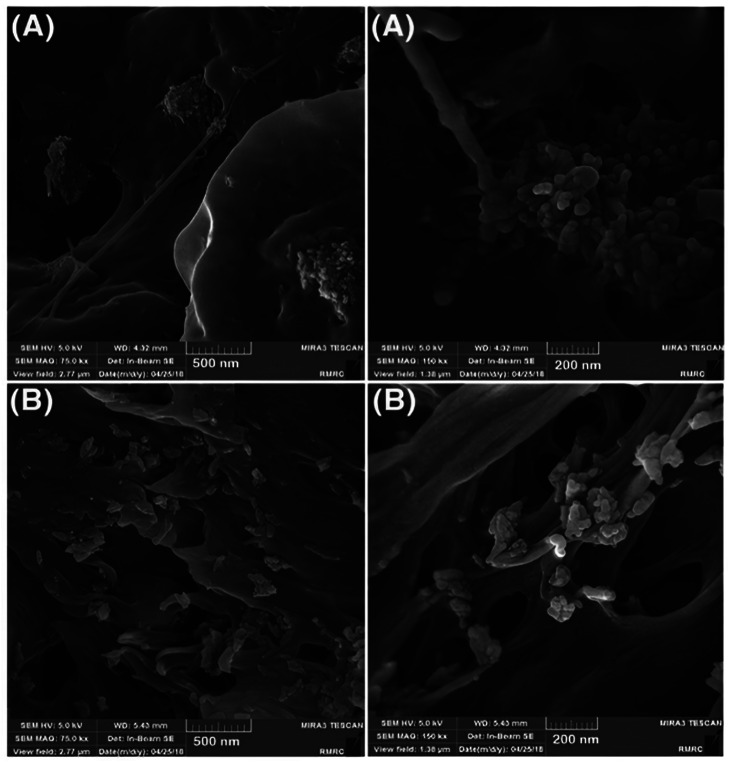
PH8 and PP4H4 cross-section FE-SEM micrographs.^251^

FE-SEM is employed to ascertain the morphological observations. At ambient temperature, the dielectric properties of pure and gelatin-blended pectin HAp were also examined for frequencies between 50Hz and 5000000Hz.^247^ FE-SEM and TEM pictures illustrated and validated HAp's morphological features.^238^ The shape of the aggregated nanoparticles created by the dual-frequency ultrasonic method was investigated using FE-SEM micrographs.^248^ Using an FE-SEM, the scaffolds' morphology was examined (FE-SEM).^249^ An FE-SEM (Model Philips Quanta FEG 250, Holland) with an accelerating voltage of 20 kV was used to analyze the morphological characteristics and features of the materials under investigation. For the micro-chemical analysis, the SEM device's attached EDS unit was used.^250^ The FE-SEM was an easy-to-use and practical instrument for researching the surface characteristics and the best way to distribute HAp nanoparticles throughout the PLA mixture. The FE-SEM cross-sectional microscopy images of PH8 and PP4H4 are shown in Figure 13. The FE-SEM images of PH8 show some noted agglomerations and the existence of nanoparticles of HAp inside its structure.^251^

### Fourier-transform infrared spectroscopy

**Fig. 14:**
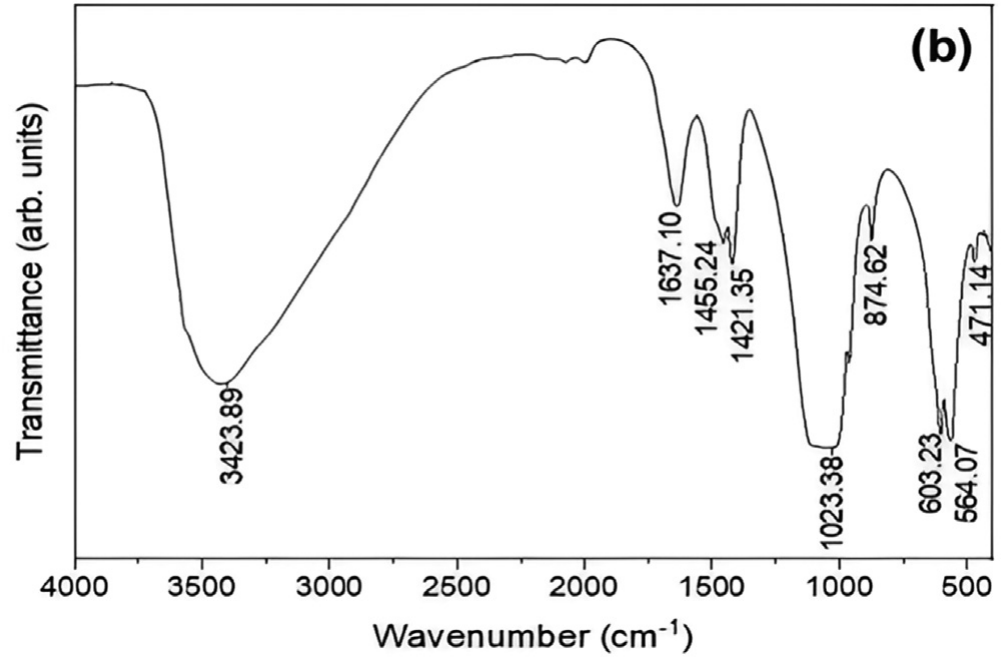
The HANPs' FTIR spectrum.^258^

Materials in the 4000-400 cm^−1^ range can be identified as polymers, organic, or non-organic, using the systematic process known as FTIR Spectroscopy.^239^ This procedure scans test samples using infrared light to observe their chemical properties.^252^ FTIR is a valuable method for determining the chemical composition of powder samples. The group consisting of phosphate (PO_4_^3−^) and the group consisting of hydroxyl (OH^−^) are the 2 groups of function that are most visible in the FTIR spectra of the produced HAp.^253^ Secondary protein structures can be detected using FT-IR. Electrostatic interactions between phosphate and amino groups and calcium ions and carboxyl groups allow proteins to bind with HA.^254^ The Amide I band is connected to the peptide bonds' C=O stretching.^255^ FTIR was used to evaluate the groups of function, molecular makeup, and bonding structure of the produced nanoparticles. Using a Perkin Elmer Frontier FTIR (PerkinElmer, USA, Spectrum400), the produced nano-HA's FTIR spectra were recorded between 400 and 4000 cm− 1.^256^ FTIR spectroscopy was used to assess the adhesives' DC. EDX mapping revealed increased calcium, phosphate, and oxygen levels in the groups of HAA-5% and HAA-10%. HAA-5% had the most significant μTBS values, followed by HAA-10%.^257^ Phosphate (PO_4_^3−^), hydroxyl (OH^−^), and carbonate (CO^2−^) groups were identified using the FTIR spectra pattern of the samples.^221^ The spectra of the FTIR were used to validate the production of HAp and revealed that the material being tested included phosphate (PO_4_^3−^) and hydroxyl (-OH) groups.^239^^,^^244^ Additional proof that the HA NPs had been effectively synthesized was supplied by the high frequencies in the spectrum of FTIR that were apparent. The principal absorption bands and the vibrating hallmark of Ca_3_(PO_4_)_2_ are shown in Figure 14. The phosphate groups in HA NPs vibrating in those modes were found as peaks at 471 cm^−1^, 564 cm^−1^, 603 cm^−1^, and 1023 cm^−1^. The absorption bands at 3423 cm^−1^ and 1637 cm^−1^Confirm the presence of water molecules associated with extremely acidic nuclear contaminants.^258^

**Fig. 15:**
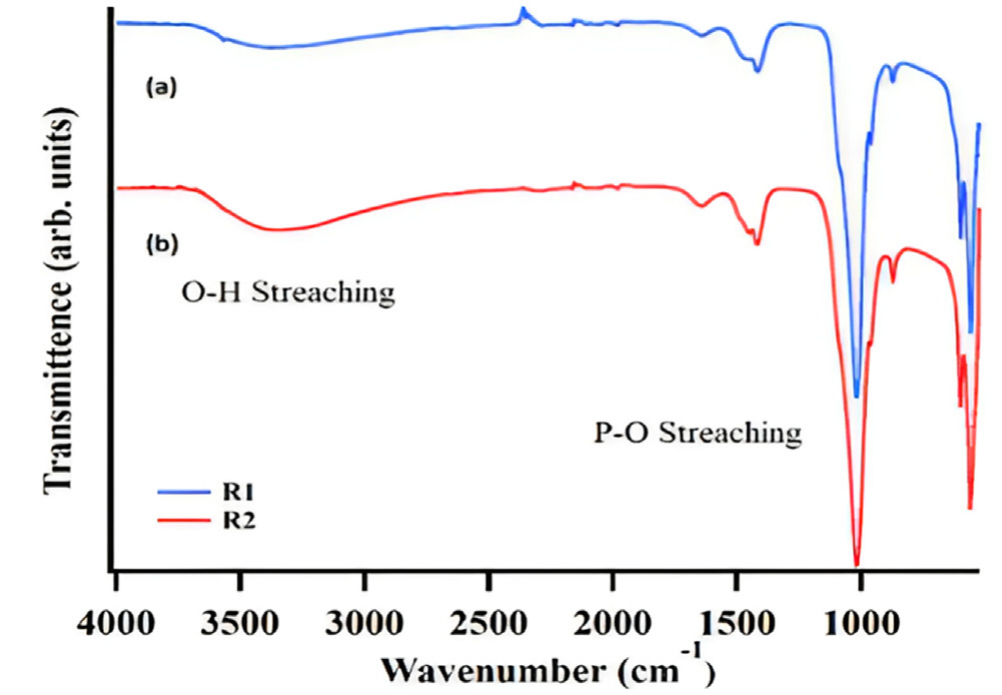
HA NPs synthesized by routes 1 (blue) and 2 (red) in its FTIR spectra.^172^

HA-Nps produced with R1 and R2 were characterized using FTIR techniques. These spectra are displayed in Figure 15.^259^

Figure 16.

**Fig. 16:**
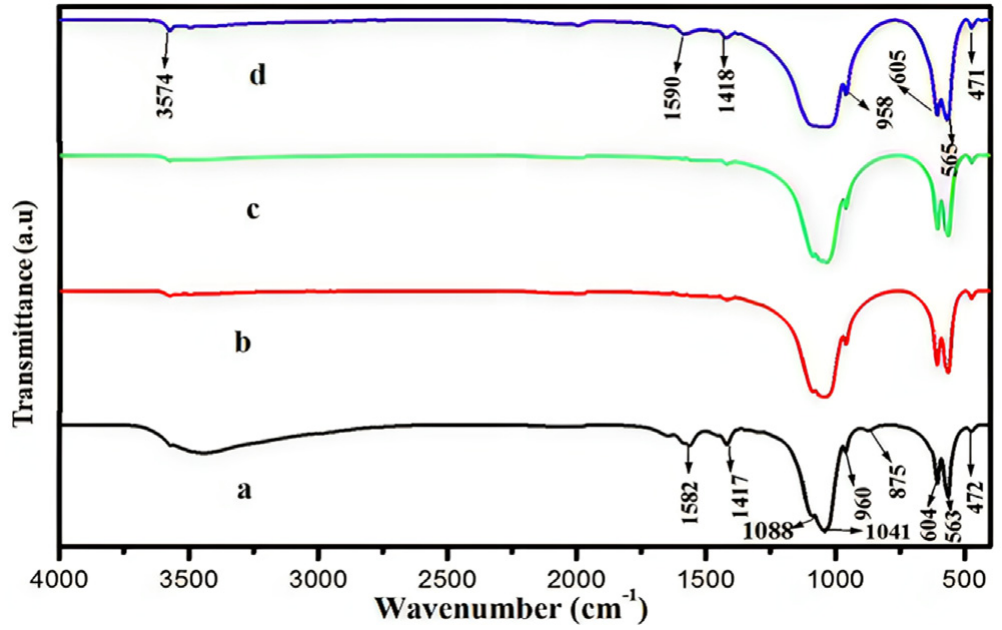
HAp's FT-IR spectra at various annealing temperatures, including (a) as prepared, (b) 700°C, (c) 800°C, and (d) 900°C.^204^

### Transmission electron microscopy

**Fig. 17:**
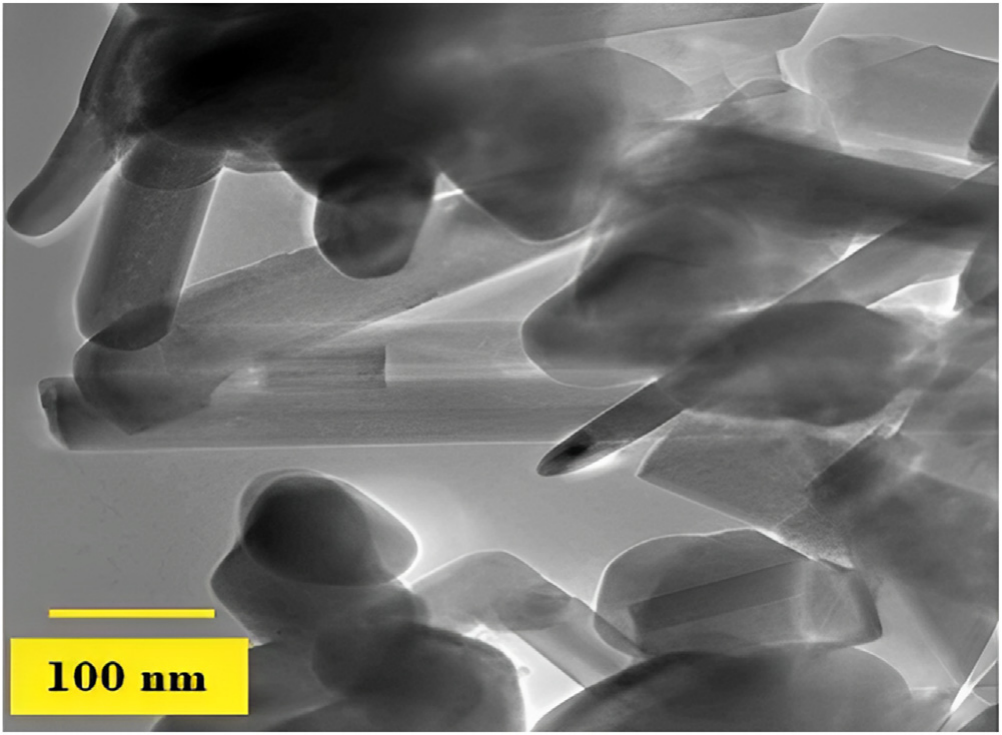
HAp nanoparticle TEM picture.^265^

By employing in situ liquid cell TEM, Wang and associates have seen the formation of Ca_3_(PO_4_)_2_ (CaP) quartz in an emulated body fluid (SBF) solution.^260^ The sizes of the crystallites were ascertained using the complementary TEM approach, and those derived using the Scherrer formula agreed well.^261^ The synthesized HAp powder's shape and chemical makeup were examined using TEM.^262^ The shape and size of the atoms in the HAp Fe composites suspension were investigated using TEM and CM 20 apparatus (Philips FEI, Eindhoven, The Netherlands) connected through a Lab6 device (which runs at 200 kV).^263^ Using a JEOL JEM-2200 FS, TEM pictures of the HAp samples were captured.^264^ TEM pictures show the presence of different-shaped particles, indicating the lack of impurity phases.^225^ Figure 17 displays the TEM picture of the microwave-synthesized HAp's recorded pattern.^265^

**Fig. 18:**
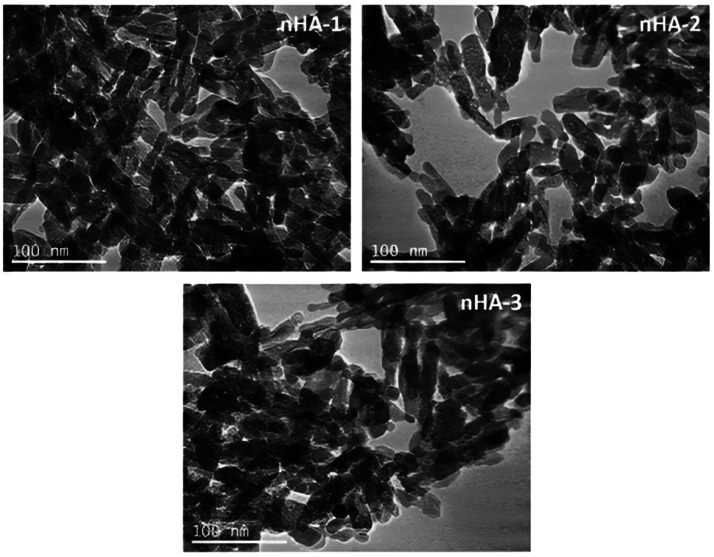
TEM images from HANPs-1, -2, and -3 show a rod-like structure with a mean length between 20 and 40 nm. The scale bar indicates 100 nm.^266^

The HANPss' size and shape were ascertained using TEM and DLS. With a mean length ranging between 20-40 nm, they had a rod-like structure. Using TEM analysis, 3 distinct nanoparticulated specimens, HANPs-1, -2, and -3, were seen. The 3 HANPs batches' TEM pictures are shown in Figure 18, together with information about their sizes and composition.^266^

### Scanning electron microscope

**Fig. 19:**
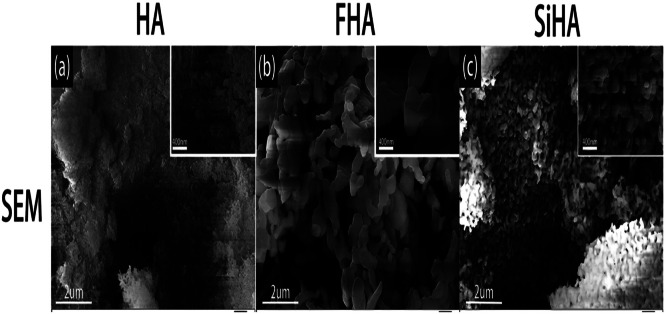
HA (A), FHA (B), and SiHA (C) SEM pictures. The precision of SEM is evident in the detailed examination of the length of HA nanoparticles, which were found to be 200 nm (Figure 8a). Similarly, the lengths of FHA and SiHA nanoparticles were examined and found to be 700-800 nm and 400-500 nm, respectively.^269^

SEM analysis is used for contrasts, shape, mineral orientation, and exploration of elements. The SEM analysis is used to analyze the morphological structure of the produced nanocomposites.^239^ The shape of phosphorus-containing (HANPs) was examined using SEM.^267^ The particles were characterized using SEM to assess the pellicle-HAp interactions. The applied particle size range exhibited significant variance, as the SEM data shows.^268^ This study examined how Si-substituted (Si HAp), Fluor-HAp (FHA), and HAp affected osteogenic differentiation, osteoclastic activity, and antibacterial characteristics. After being created using a sol-gel technique, HA, FHA, and SiHA were characterized using SEM. A SEM was used to study the materials' surface characteristics. Figure 19 displays the SEM results for 3 distinct types of rod-shaped nanoparticles but with varied sizes.^269^

**Fig. 20:**
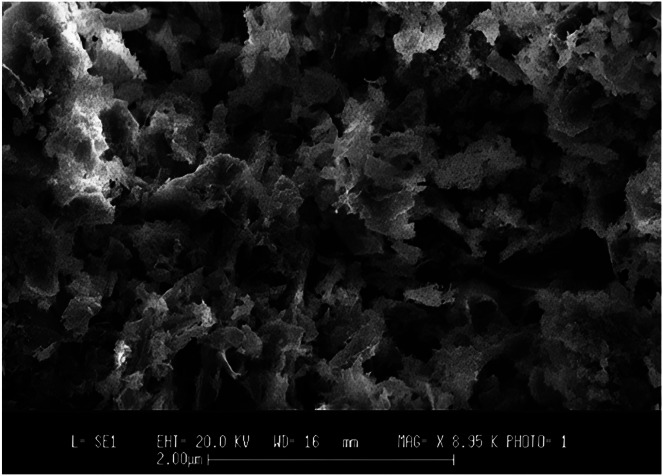
A picture of calcined (HAp) obtained using SEM.^262^

An SEM (SEM: Quanta 400) was employed to comprehensively analyze the microstructures and morphologies of the HAp crystals.^245^ The synthesized HAp appeared crystalline and compact based on the SEM morphologies.^240^ The appearance and microstructure of the generated HAp nanoparticles were examined using a (Phenom-World X, Netherlands).^270^ SEM examined hydrogels' shape concerning their HA NPs content.^271^ An SEM image was taken to inspect the synthesized HAp's form (Figure 20).^262^

## Advantages of hydroxyapatite nanoparticles in dental implants

In dentistry, nanoparticles offer a wealth of potential for study and a wide range of applications. Various types of nanocoating can be extensively used to eHANPsnce the structural integrity of teeth. The ongoing research and development of more biocompatible materials could potentially prevent dental implant failure, a current area of significant research interest.^272^ Nanoparticles closely mimic the texture of natural teeth, and their adaptability to fit ay shape provides a sense of reassurance about their versatility.^273^, ^274^

Around the year 2000, advances in nanotechnology significantly impacted materials science, oral surgery, diagnostics, and restorative dentistry, and the profession of nanodentistry began to take shape. The goal of nano dentistry is to provide patients with complete dental healthcare. Because of the development of more sophisticated and precise diagnostic techniques, many oral disorders can be prevented or treated early in life.^275^, ^276^

**Fig. 21:**
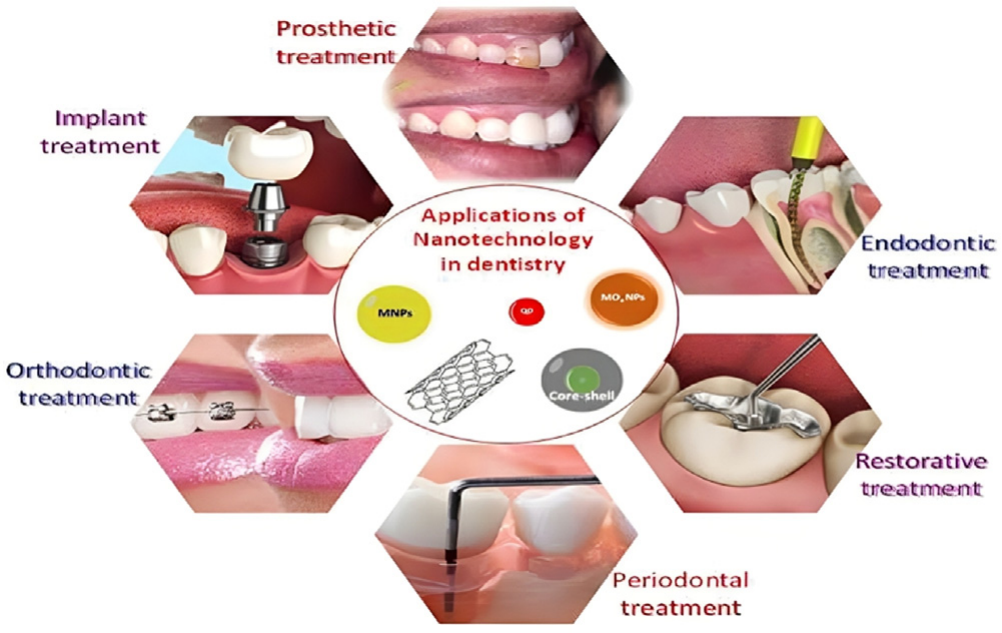
Nanomaterial applications throughout various dental specialties.^277^

Figure 21 illustrates the effective use of nanomaterials in dentistry for various procedures, including prosthodontic, endodontic, restorative, periodontal, orthodontic, and implantology treatments.^277^

HAp, a ceramic biomaterial, has demonstrated good bioactivity, biocompatibility, osteoconductive properties, nontoxicity, and non-inflammatory properties in both vitro and in vivo.^278^

**Fig. 22:**
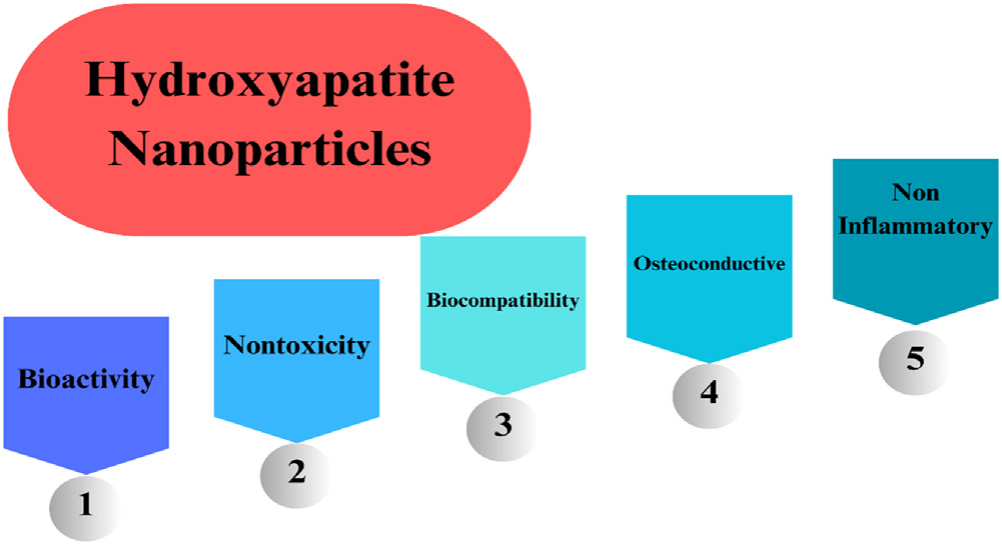
Nature of HAp.

**Fig. 23:**
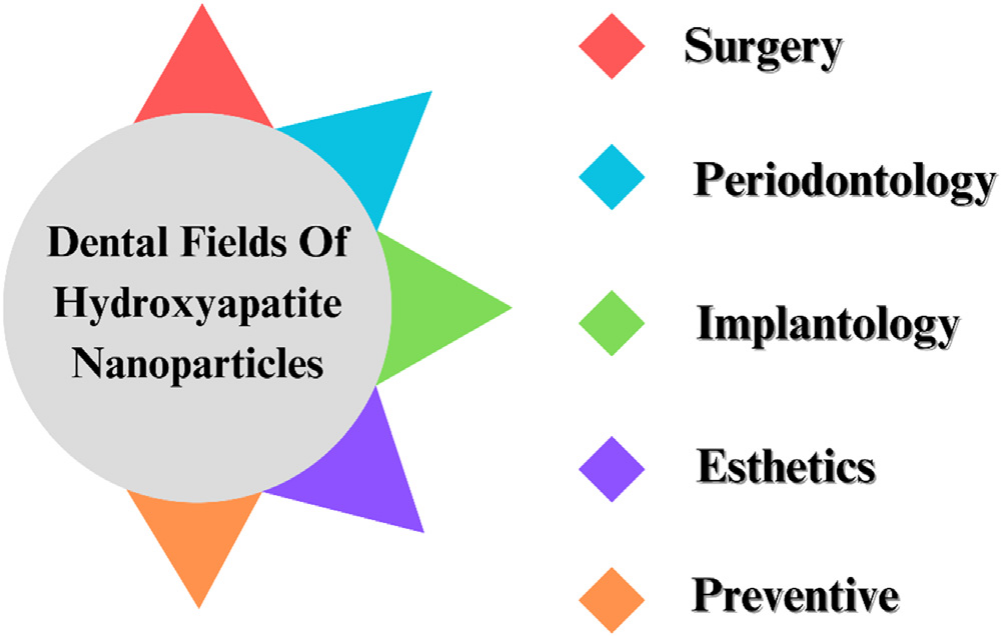
Dental fields of HANPs.

**Fig. 24:**
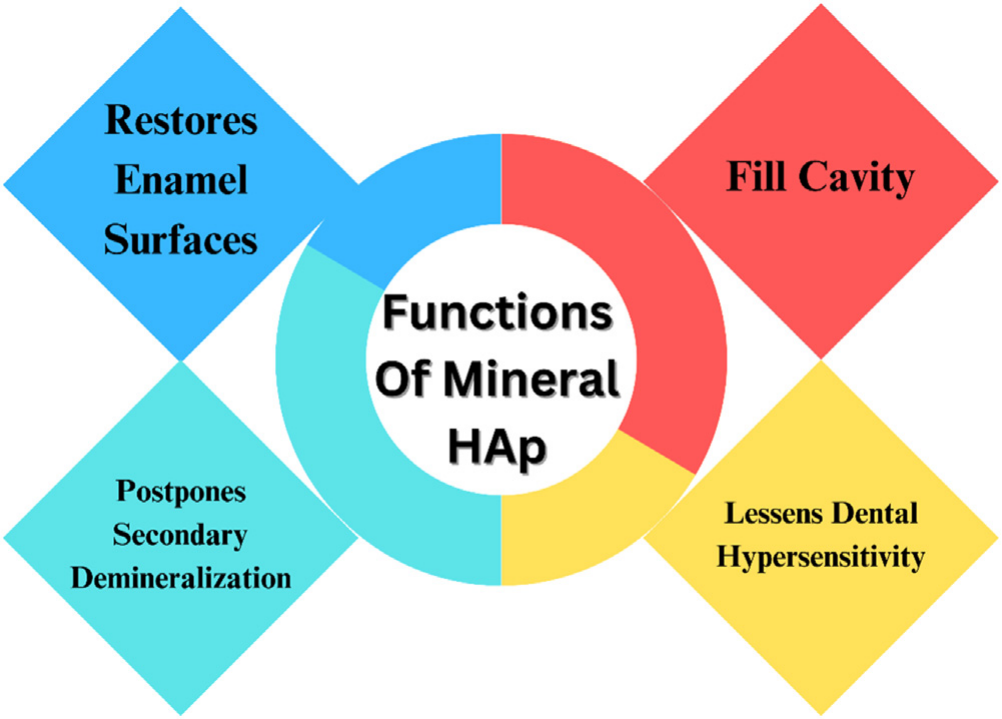
HANPs as a mineral.

Figures 22, Fig. 23, Fig. 24

Because of its biocompatibility, HAp —the primary component of the mineral portion of teeth and bones—is a biomaterial that has been researched the most in the medical and dentistry fields. Over the past ten years, research on HA NPs, n-HAp, has focused on oral implantology, bone rebuilding, restorative dentistry, and preventative dentistry.^279^ Because (HA NPs) may target specific organs and minimize adverse effects, they can be employed as medication carriers.^280^ Over the past ten years, HA NPs and n-HAp research have been conducted in oral implantology, bone restoration, restorative dentistry, and preventive dentistry. Significant remineralization of early enamel lesions can be achieved with HA NPs. These nanoparticles have also been used as an additive to eHANPsnce, a widely used dental material used mainlyin preventative restorative and regenerative dentistry.^279^ Optimizing the precise molecular mechanisms by which Hydroxyapatite Nanoparticles (HANPs) promote remineralization in the early stages of dental caries requires molecular-level research into the compounds' interactions with the dental work matrix. By tailoring the size, shape, and surface characteristics of HANPs that they interact with, it may be possible to enhance the transport and deposition of calcium and phosphate ions while also facilitating their penetration and attachment to the enamel. Furthermore, HANPs may benefit from surface functionalization by adding bioactive substances like growth factors and peptides to increase their remineralizing effectiveness. The use of sophisticated characterization methods, including atomic force microscopy (AFM), transmission electron microscopy (TEM), and energy-dispersive X-ray spectroscopy (EDX), might clarify the processes by which HANPs encourage mineral ion deposition and crystal development. It may be possible to improve the efficacy of HANPs in clinical settings and guide treatments aimed at managing dental cavities by comprehending the dynamics of this process.^281^, ^282^, ^283^ Because (n-HAp) is biocompatible and resembles the structure of nonorganic bone, it is a material with various applications. It is applied in several dental fields, including surgery, periodontology, implantology, esthetics, and preventive.^284^

Most studies in this sector focus on in vitro designs, ignoring the interactions between salivary pellicle and apatite. There are no publications about the effects of HAp on other dental materials.^285^ According to a recent literature study by Epple M., HAp particles are a harmless and nonimmunogenic substance that has no negative impact on human health when used in appropriate dosages.^286^ HAp mineral fill cavity lessens dental hypersensitivity, postpones secondary demineralization, and restores enamel surfaces. Tooth tubules can readily accept HAp nanoparticles. Comparable in composition to bone and tooth, biocompatible, clings to dental enamel, repairs periodontal defects, and shields the tooth by encasing it in a layer of false enamel. They can bind with proteins to produce protein-particle complexes, which are then eliminated by macrophages within the tissues. These particles are transported to and dispersed by blood throughout the liver, spleen, and lungs. Oxidative stress, the signaling system, and the inflammatory response can all be impacted by nanoparticle toxicity.^287^

**Fig. 25:**
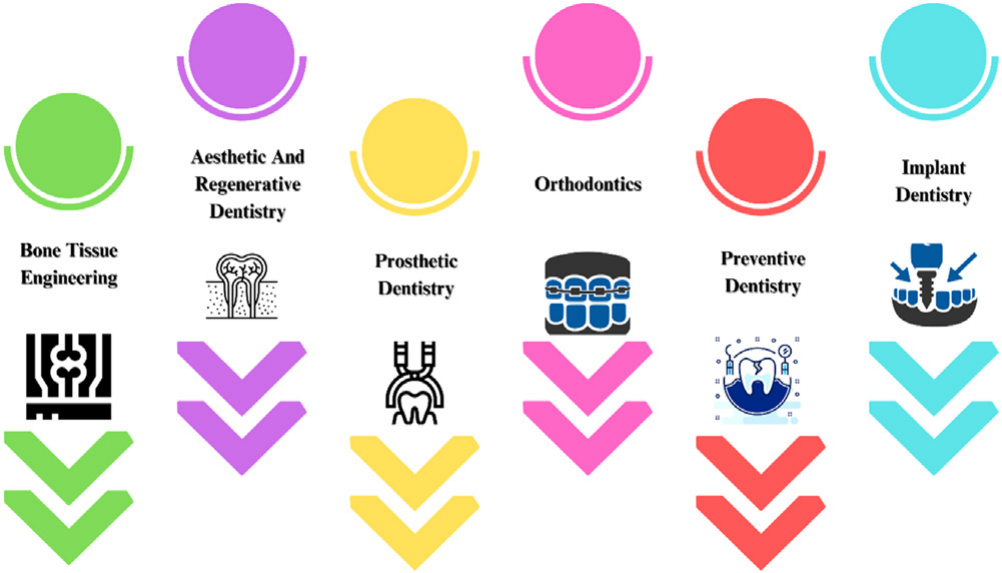
The application of HA NPs in many dental specialties.^282^

HAp is the principal mineral found in teeth and bones. About 65% of the weight of bone is made up of HAp, a needle-shaped substance with a length of 60 nm and a width of 5-20 nm that ensures the structure's stiffness and strength.^288^ HAp crystals comprise most of the enamel and dentine layers in the tooth's construction. Whereas the dentin layer is made up of 70 weight percent inorganic matrix, 20 weight percent organic matrix, and 10 percent water, the enamel layer is made up of 96 weight percent inorganic matrix, organic elements (such as proteins and lipids), and 4 weight percent water.^289^ According to our research, dentistry and oral and maxillofacial surgery have given HAp and HA NPs a great deal of attention in the past ten years (Figure 25).^282^

**Fig. 26:**
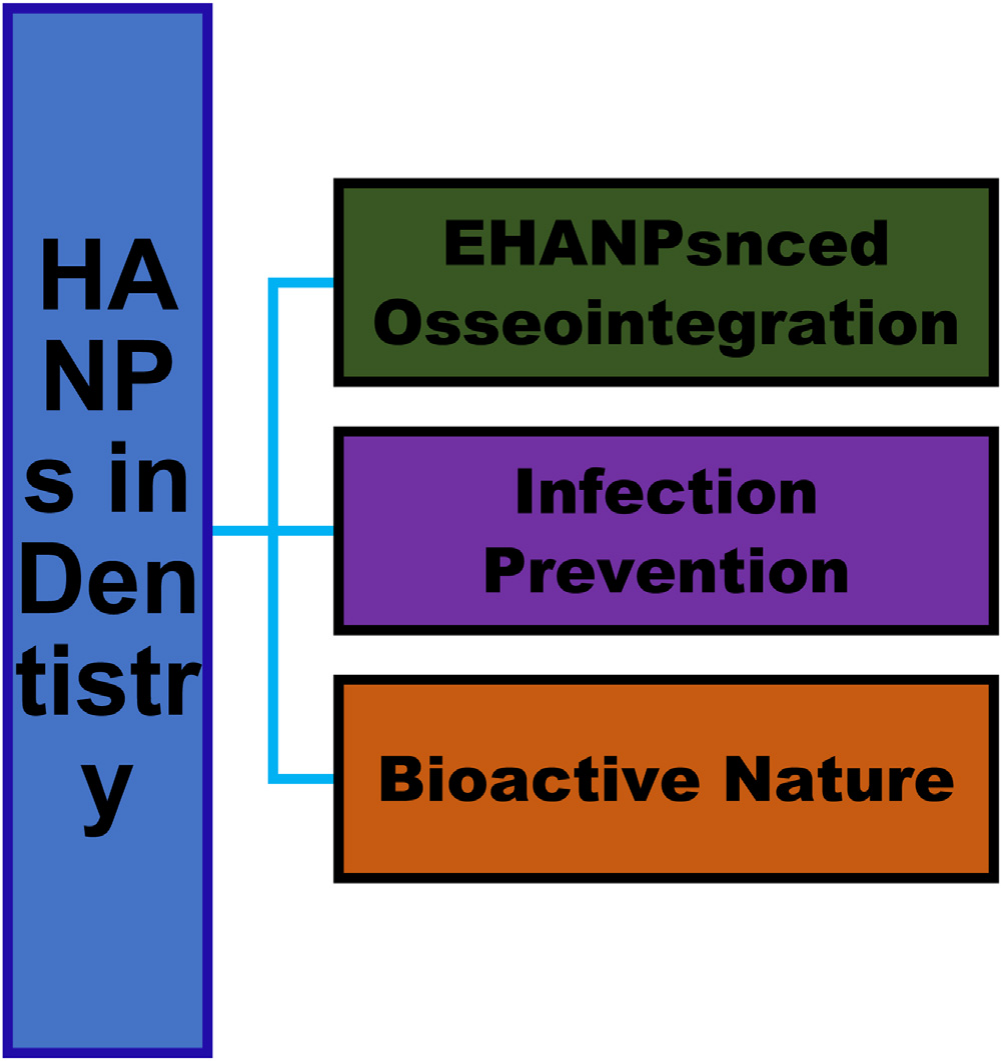
Importance of HA NPs in dentistry.

**Fig. 27:**
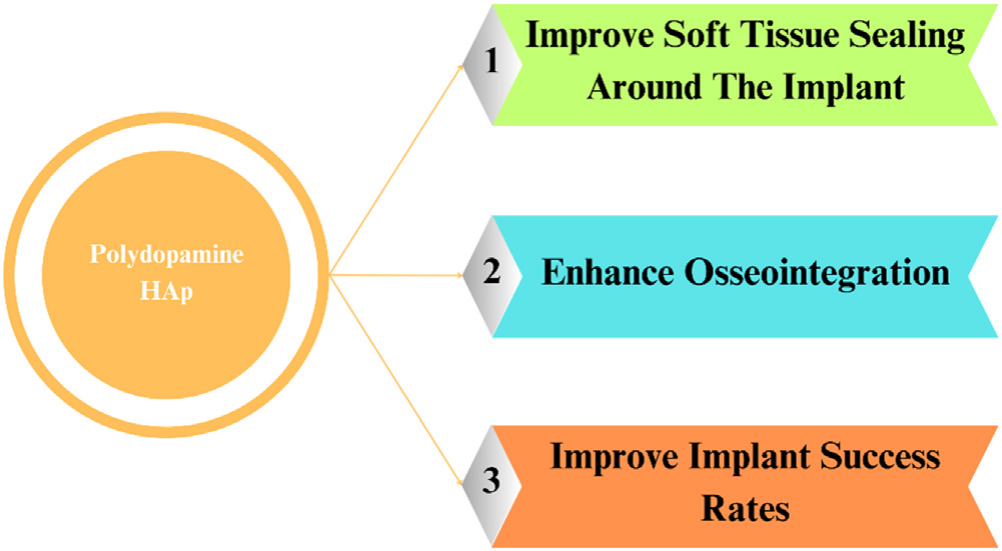
Uses of (PHG) coating polydopamine HAp.

**Fig. 28:**
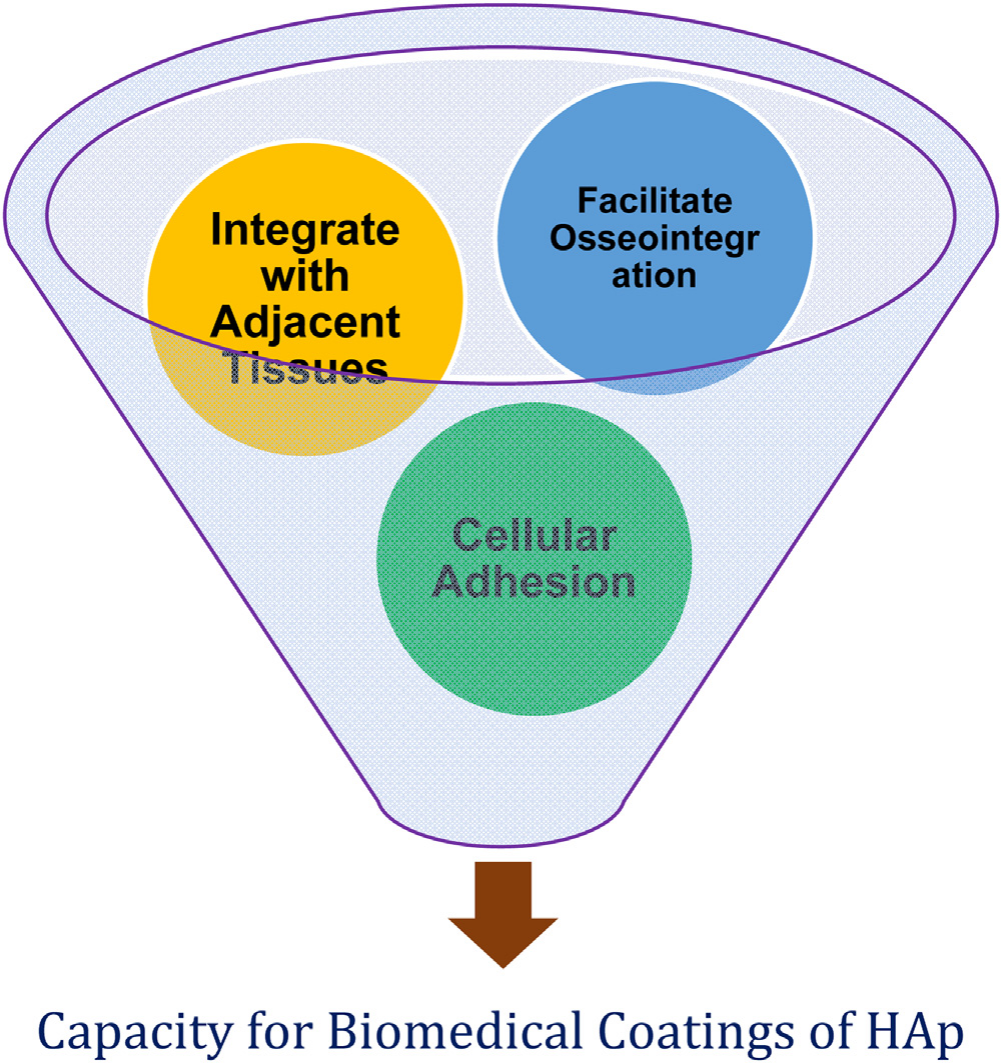
Graphical view of HA NPs in biomedical coating.

**Fig. 29:**
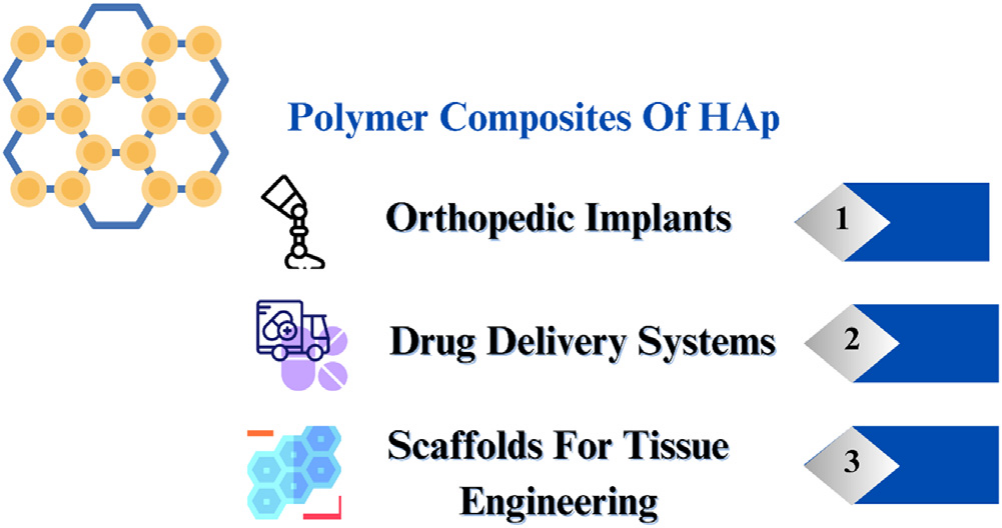
Different types of polymer composites of HANPs.

**Fig. 30:**
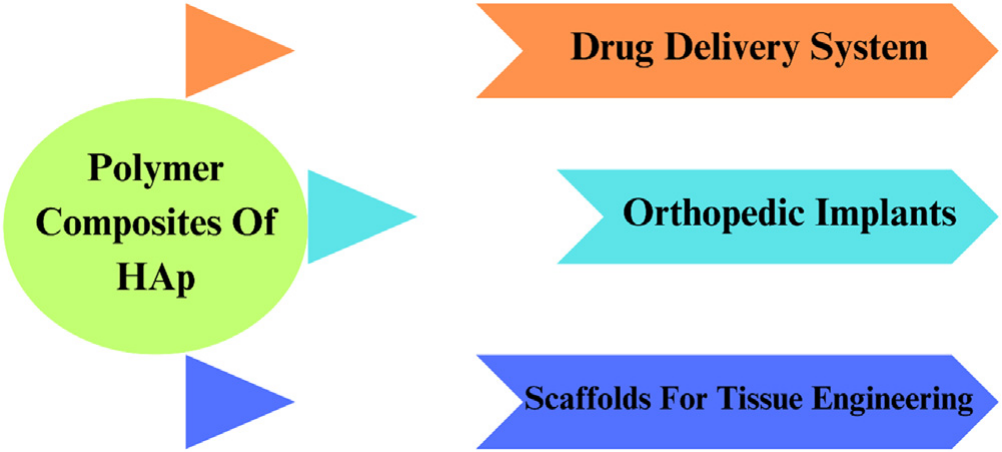
Different uses of polymers of HAp.

Here is a graphical representation of the significance of HA NPs in dental implants:

Fig. 26, Fig. 27, Fig. 28, Fig. 29, Fig. 30

### eHANPsnced osseointegration

The early osseointegration of oral implants can be influenced by surface features such as chemical composition, mechanical elements, and micro- and nano-roughness.^290^, ^291^ Dental implants are prosthetic devices that an orthodontist surgically inserts into a patient's jawbone to replace lost teeth roots. The maxilla or mandible merges with the dental implant's surface following placement. This phase, known as “osseointegration,” is crucial for guaranteeing the long-term functionality of dental implants and averting implant malfunctions.^292^ According to Dorland's Dictionary, the process of “direct anchorage of an implant by formation of bony tissue around the implant without the growth of fibrous tissue at the bone-implant interface” is known as osseointegration. The phrase is subsequently modified to read, “direct structural and functional association between the surface of an implanted bone that bears weight and living, ordered bone.”^293^

Dental implants have advanced thanks to nanotechnology. It helps to comprehend and accomplish cell-specific tasks; as a result, crucial osseointegration phases can be controlled by altering the implant surface at the nanoscale.^294^ Especially in directed bone regeneration, Osseointegrated implants are aesthetically pleasing materials used for many years in oral and maxillofacial surgery.^295^, ^296^, ^297^ Implant failure may occur from inadequate osseointegration and antibacterial activity caused by the intrinsic biological inertness of the titanium surface. The most frequently utilized substance in the biomedical industry is HAp. Its structural and chemical resemblance to genuine bone makes it one of the bioactive coating materials.^298^ To speed up their osteointegration, more work is being put into creating innovative and active surfaces, like nanosized crystalline HAp coating.^299^ A paper proposes a composite multifunctional coating (PHG) prepared using gelatin and polydopamine/ HA NPs to improve soft tissue sealing around the implant, eHANPsnce osseointegration, and implant success rates. It also investigates the effects of this novel coating on cell adhesion, proliferation, antibacterial activity, and osteogenic differentiation and evaluates its immune-related properties.^300^

HAp can be deposited on a Ti alloy surface using various deposition techniques to improve their osseointegration with human bone.^301^ Dental implants with different surface roughness and alterations, such as those that have been plasma-sprayed, acid-etched, blasted, oxidized, coated with HAp, or a combination of these processes, are frequently placed by clinicians. Surface alterations are intended to promote early osseointegration and guarantee long-term implant-bone contact without significantly reducing the amount of marginal bone loss.^302^, ^303^ Dental implants are coated with nanocomposite to eHANPsnce their compatibility with natural bone. They are utilizing HAp and other chemical and physical techniques. "Biocompatibility and osseointegration can be improved by using secondary nanoparticles," the researchers concluded in a clinical investigation.^304^ Different materials, such as BMP-2, HAp, and antimicrobial compounds, eHANPsnced implant integration and surface modification. By eHANPsncing osseointegration and preventing implant infections, these coatings raise the implant's success rate.^305^ At first, noted for its remarkable biocompatibility and bioactivity, HAp rose to popularity in dentistry and bone tissue engineering. It was a perfect fit for various biomedical coatings and implants because of its capacity to induce cellular adhesion, facilitate osseointegration, and integrate with adjacent tissues.^306^

HAp encourages osseointegration, or the integration of the implant with the surrounding bone, in orthopedic and dental applications.^307^ Polymer-HAp Composites: The mechanical characteristics, bioactivity, and biocompatibility of composite materials can all be improved by incorporating HAp nanoparticles or microparticles into polymer matrices.^308^ In HAp-polymer composites, polymers like Polylactic Acid (PLA), Polyglycolic Acid (PGA), Polycaprolactone (PCL), and gelatin are frequently utilized as matrices.^309^

These composites are used in orthopedic implants, drug delivery systems, and scaffolds for tissue engineering. The HAp component encourages osseointegration and bone regeneration, while the polymer offers mechanical support.^310^

**Fig. 31:**
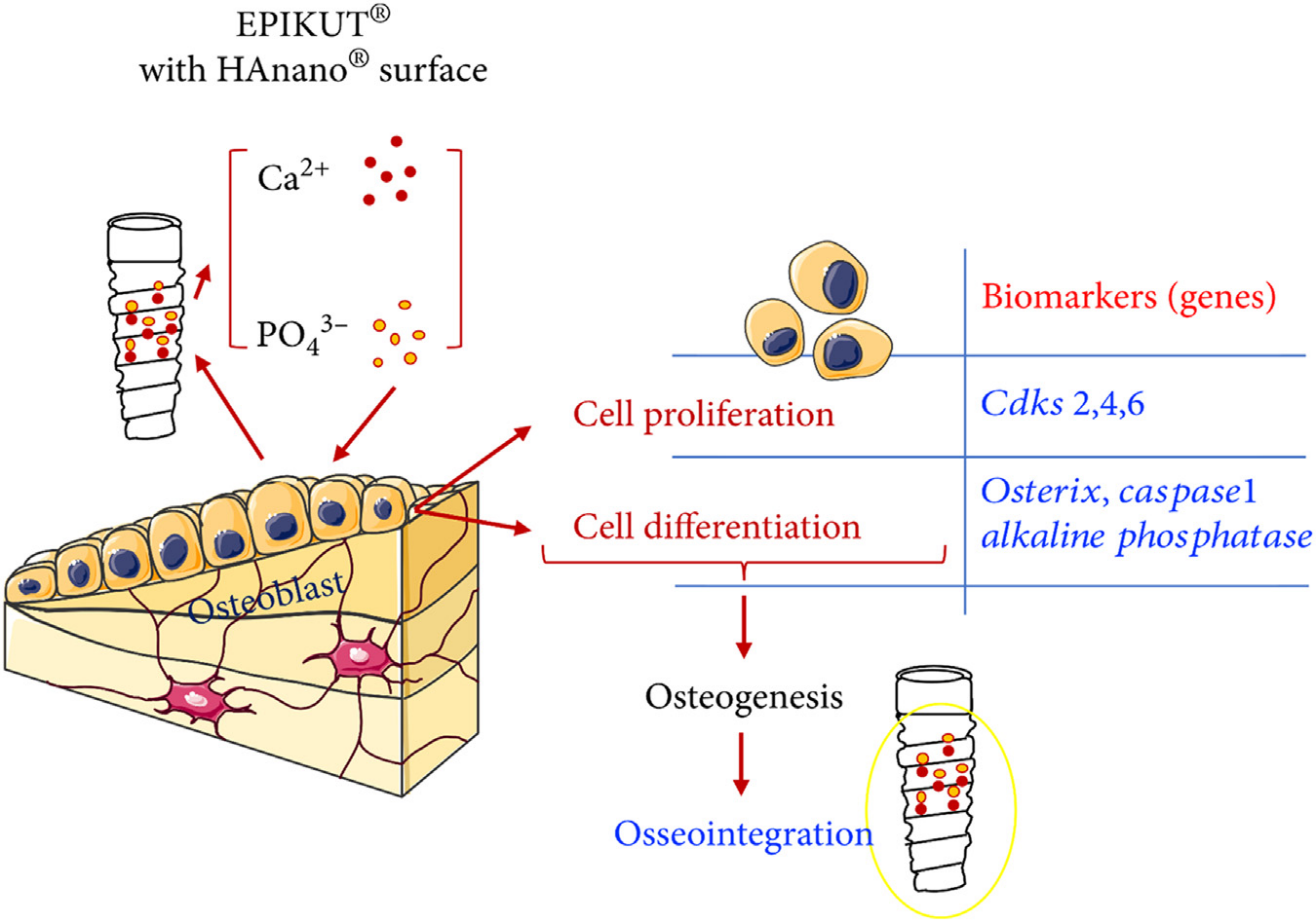
Summary of the molecular processes that the Hanano surface in osteoblasts initiates. The primary biological pathways that the HAnano-modified surface initiates in osteoblast responses are illustrated in this system. Osteoblasts that come into contact with the surface at early 3 and 24 hours upregulate the activity of a collection of genes linked to cell adhesion, further impairing the expression of genes related to osteoblast differentiation. Crucially, it is proposed in the literature today that the release of calcium and phosphate ions activates an upstream signaling pathway that promotes osteoblast development and proliferation. These phases of osteoblast biology collectively result in the osteogenesis process, which is anticipated to be recapitulated during the osseointegration of dental implants.^299^

HAp coatings are frequently utilized to increase metallic implants' osseointegration and biocompatibility, lowering the chance of implant failure and eHANPsncing long-term stability.^306^ Thermal and plasma spraying methods providethe extra benefit of resisting biocorrosion and rapid degradation of Hydroxyapatite nanoparticles.^311^ The coating of hydroxyapatite nanoparticles has characteristics similar to those of natural bone. It shows the same mechanical stability in periodontal disease.^312^ By promoting a chemical interaction with bone, dental implants coated with hydroxyapatite nanoparticles can increase osseointegration, biological fixation, and stability.^313^ For this reason, the biological effect of these nanoparticles will facilitate the post-effect of dental loss. During the process of osseointegration, the coating methods of dental implants with HAp directly impact the calcification and cell differentiation of the bone matrix.^314^ An overview of the study's key conclusions is shown in Figure 31, which indicates that Hanano® offers osteoblasts a suitable microenvironment and surface to reprogram a set of genes that control the osteoblasts' adhesion, proliferation, and differentiation, all of which lead to the osteogenesis process. During the osseointegration of dental implants, these steps of osteogenesis are replicated in an appositional bone development way.^299^

The primary ongoing difficulty in implant dentistry is achieving quick osseointegration in low-density bone, an area where nanostructured HAp surfaces emerge as a possible solution.^315^ Finally, we have demonstrated that the Caspase1 gene was also significantly upregulated in response to Hano®. This implies that the inflammasome may play a role in the osteoblast differentiation mechanism, as previously proposed, and that Hano may also act as a stimulus for osteoclast genesis, as indicated by the increased Rankl/Opg ratio in osteoblasts. Further research is necessary to determine the effectiveness of Hanano on the biology of osteoclasts.^315^, ^316^

### Bone regeneration

Due to a variety of bone illnesses, including infections, tumors and the resulting fracture, birth deformities, and bone loss from trauma, explosions, or accidents, bone regeneration is significant globally.^317^ To get over this problem, HAp, which has good biocompatibility and biodegradability, is typically combined with PEEK to generate strong bone bonds with bone tissue (Nga et al.^318^ Injuries and abnormalities in the bone are challenging problems for regenerative medicine. Biomaterials are used as scaffolds in Guided Bone Regeneration (GBR) procedures to promote the healing of injured bone tissue.^319^ HAp is the primary inorganic-organic composite substance that makes up bone.^320^, ^321^ HAp is Indonesia's most commonly utilized bone grafting material to prevent bone loss after tooth extraction.^322^ But HAp deteriorates gradually over around 24 months.^321^, ^322^, ^323^, ^324^ The secret to speeding up bone regeneration and repair during the bone repair process is to preserve osteoinductivity in the region of the bone defect. One effective technique is bioactive molecules, such as the local administration of osteoinductive growth factors.^325^ Because HAp nanostructures are effective osteoconductive, bioactive, and biocompatible materials that improve bone adherence, they are primarily used in medicine to cure bone defects.^326^ Chemically speaking, HAp is comparable to the inorganic portion of human bone and teeth.^327^, ^328^, ^329^ A composition akin to the mineral bone (HAp) has been applied to microporous scaffolds to promote bone development.^330^ Hydroxyapatite nanoparticles (HANPs) can adherely improve the long-term integration of the implant and bone when applied to various implant materials. According to this study, titanium implants coated with HA had survival rates similar to those of uncoated implants, suggesting that HA coatings may promote osteointegration. A deeper investigation is required to determine the long-term impacts of these combinations. However, applying HA top layer to other materials, including stainless steel, has enhanced biocompatibility and may influence bone response.^331^, ^332^

Over the past ten years, research on HA NPs, known as n-HA, has focused on oral implantology, bone rebuilding, restorative dentistry, and preventative dentistry. The application of HA NPs as an additional material has improved commonly used dental materials, primarily in preventive but also in restorative and regenerative applications. These nanoparticles have been shown to have considerable remineralizing effects on early enamel defects. Nowadays, HAp is used in clinical oral surgery, particularly in the regeneration of bone tissue. It can treat periodontal bone defects, fill in bone defects after cyst removal and apicoectomies, or widen atrophic alveolar ridges when dental implants are removed. Furthermore, HAp scaffolds are employed in pre-prosthetic surgery to broaden the alveolar ridges or in maxillofacial surgery to repair portions of the maxillary bones or other facial skeleton.^279^

The long-term usage of doped hydroxyapatite nanoparticles (HANPs) in dental and orthopedic implants would have cytotoxic and safety implications depending on the kind and concentration of dopant. While medications containing metals like iron, zinc, and silver may improve mechanical characteristics and bioactivity, they may also have harmful side effects. High quantities of certain metals, for instance, might cause toxicity, inflammation, or unfavorable biological reactions that could impair implant lifetime and integration. Therefore, to ensure that doped HANPs are safe and efficacious for clinical application, rigorous research into their synthesis, chemistry, and toxicity is required.^333^

Fig. 32, Fig. 33.

**Fig. 32:**
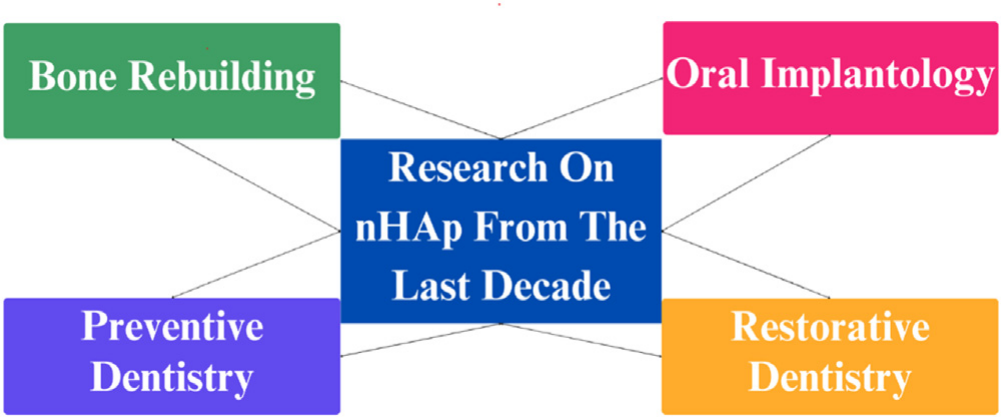
Research on HANPs from the last decade in different sectors of dentistry.

**Fig. 33:**
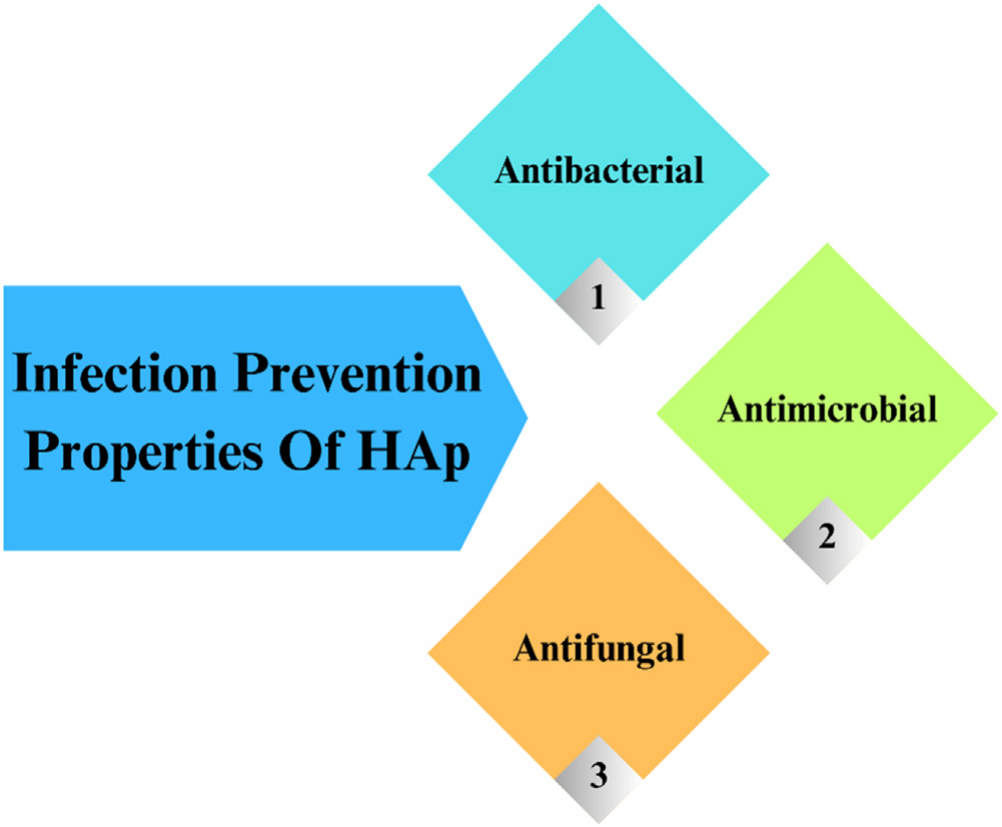
Infection prevention properties of HAp.

Tschoppe et al. examined the impact of various HA NPs toothpaste on the remineralization of dentine subsurface lesions and bovine enamel in an in vitro investigation compared to amine fluoride toothpaste. According to their findings, HA NPs toothpaste had comparable abilities to remineralize subsurface lesions in dentine and enamel. Furthermore, fluoride toothpaste had the most negligible effect on remineralization of all the hard tissues tested, yet deeper lesions have also been reported.^334^ By offering a surface that can draw and hold onto protein and cellular adhesion to increase implant durability, hydroxyapatite (HA) coatings on dental implants promote osseointegration. However, questions surround these coatings' long-term endurance, especially when subjected to constant mechanical stress. Not all coatings exhibited coupling with the substrate, which might result in wear or delamination if the coating was poorly adhered to the substrate or the mechanical stresses were strong enough to surpass the coating's tensile strength. HA coatings are thought to protect first, but studies reveal that mechanical stress over time may shorten their lifespan and increase the chance of failure. Further investigation is required to evaluate the effects of varying loading circumstances on the coating integrity and wear or delamination tolerance of HA-coated implants to evaluate their performance under ongoing mechanical stress. Several variables, including the coating's thickness, the bonding method used, and the mechanical characteristics of the implant material below, affect these results.^335^, ^336^, ^337^ One of the bioceramics frequently employed for bone regeneration is HAp nanoparticles (HA NPs); these particles dissolve more quickly than HAp microparticles, but their pore size is insufficient.^338^ In a mouse model of vertical bone augmentation, an in vivo investigation was carried out to assess the effectiveness of HAp -HAp-containing bone graft gel in stimulating bone regeneration.^339^ A kind of bioceramics made of HAp consisting of a micro-whiskered scaffold (wHA) supplemented with many layers of releasable HANPs. To maximize the loading quantity of HANPs for osteoporotic bone production, these innovative bioceramics (HA NPs) can be tuned.^340^ Our lab initially observed that sick osteoblasts isolated from osteoporotic bone explants might be encouraged to produce bone by low-crystallinity HA NPs with a size of 40-60 nm.^341^

HAp offers a wide range of uses in dentistry and other problematic tissue issues because it is biocompatible, bioactive, has a low solubility in water, and resembles the mineral portion of hard tissue in humans. When fluoride ions, which are present in bone and enamel, are combined with HAp, they lessen the solubility of the latter and retain a similar level of biocompatibility—A major contributor to bone regeneration.^342^ Research results showed that the biomimetic HAp microsphere can improve the regeneration of alveolar bone.^343^ These days, HAp is used in clinical oral surgery, particularly in the regeneration of bone tissue. It can treat periodontal bone defects, fill in bone defects after cyst removal and apicoectomies, or widen atrophic alveolar ridges when dental implants are removed. Furthermore, HAp scaffolds are employed in pre-prosthetic surgery to broaden the alveolar ridges or in maxillofacial surgery to repair portions of the maxillary bones or other facial skeleton.^344^, ^345^ Biomimetic HAp nanoparticles (HA NPs) offer superior biological features such as biocompatibility, non-toxicity, and favorable reactions from the immune system and inflammation during the remineralization of the enamel surface.^346^ Numerous studies demonstrated that adding HANPs evanesced the effectiveness of various materials for both in vivo and in vitro bone repair.^347^

Because of its exceptional biocompatibility, HANPs are a promising material for various biological applications, including bone regeneration and dental uses. One of the most essential biomaterials, HAp, makes up a sizable portion of the mineral layer of teeth and bone. Compared to traditional fluoride, the remineralizing effect produced and provided by HANPs is more beneficial for bone regeneration. The qualities of HANPs are sufficient for bone remineralization and tissue growth, maintaining the tissues' health and improving resistance to sensitivity and various forms of stress or strain. Finally, when it comes to the regeneration of bones in different areas of the human body, the usage of HANPs can play a significant role. This regeneration can occur more quickly and effectively with the correct application of HANPs and the most applicable strategies and approaches.^326^

### Infection prevention

The insertion of a foreign substance into the body is typically linked to bacterial colonization and implant-related infections (peri-implantitis), which can result in severe consequences and eventual failure despite all the advancements in biomaterials.^348^ Medical equipment can be shielded from host immune response and antibiotics by a biofilm layer formed by certain gram-positive and gram-negative bacterial strains.^349^, ^350^ Dental implants coated with nanoparticles can have antibacterial qualities, reduce the risk of peri-implant infections, and encourage soft tissue attachment, all of which increase the longevity of the implants.^351^ Antibacterial HAp spherical granules with varying MgO percentages were created in a study to be utilized as bone substitutes and guard against orthopedic and dental infections. Even though HAp performs exceptionally well in bone repair, it cannot stop bacterial colonization alone. Developing novel bone grafts with antimicrobial qualities is crucial to prevent severe infections following implantation.^352^ The work aimed to prepare and characterize HAp nanoparticles doped with ions such as Ni2+, Sn2+, and Mo^3+^ in vitro to prevent infections, particularly in bone tissue engineering.^353^

#### Antibacterial

HAp is a substance that finds numerous applications in biomedicine, including antibacterial applications, orthopedic dentistry, and drug delivery.^354^ HAp's crystalline structure and composition can accommodate a range of ionic replacements, each with unique benefits like osteoinduction or antimicrobial activity.^355^ Both polymeric matrix and HAp are employed as carriers to increase their effectiveness against different pathogenic species. These composite materials show promise as antimicrobial coatings for orthopedic and dental implants and potential surgical bone cement.^356^ Using the disk diffusion method, HAp composites (HA-Ag NPs) showed good antibacterial efficacy against 2 bacterial strains (Escherichia coli and Staphylococcus aureus) in the investigation.^357^ The CS/Ag/HA coatings offer strong antibacterial qualities against both Staphylococcus epidermidis and Escherichia coli, according to the results of antibacterial tests. The Osteoblasts (OB) culture demonstrates the excellent biocompatibility of the CS/Ag/HA coatings.^358^ Many different biocompatible materials have been used as coatings or beads to enable antibiotic release. One of the components is artificial HAp.^359^, ^360^, ^361^

#### Antimicrobial

Since these biofilms are frequently resistant to conventional antimicrobials, implant coverings made of nanoparticles may provide a long-term solution for infection prevention.^362^ Since HAp is employed as an implant coating material, there is little data on its cytotoxicity and antimicrobial activity but none on its potential antibacterial mechanism.^363^ Researchers created and assessed HAp-gelatin/curcumin nanofibrous composites to ascertain their antibacterial efficaciousness against Streptococcus mutans, Staphylococcus aureus, and Escherichia coli.^364^ Antimicrobial efficacy against *Streptococcus mitis* and *Streptococcus gordonii*, the early colonizers of supra-gingival plaques, was made possible by the HAp-coated Ti. The hydrophilicity of the surface may be the reason for this antibacterial activity, which creates a repulsive force between the bacterial cell membranes and the Ti surface coated with HAp.^365^

### Bioactive nature

One of the most important developments in contemporary biomaterial science and research is the presence of bioactivity in dental resin composite restorations.^279^ In addition to being highly biocompatible, the implant surface needs to be bioactive and capable of triggering osseointegration.^301^ The bioactivity of HAp was significantly increased with the insulin administration with HANPs.^366^ HAp is a typical bioactive substance utilized in clinical and biological applications.^367^ In contrast, researchers have been drawn to HAp due to its evanesced bioactivity and biocompatibility.^368^ Furthermore, it is anticipated that nano-HAp will have more excellent bioactivity than coarser crystals.^369^ Because of its strong biocompatibility, osteoconductive, bioactivity, non-inflammatory, non-immunogenic, and non-toxic nature, it has potential qualities for use in orthopedic procedures and dental implants.^370^, ^371^, ^372^, ^373^, ^374^, ^375^, ^376^, ^377^ HAp can enhance the qualities of materials now utilized in restorative dentistry because of their excellent biocompatibility, bioactivity, and antibacterial impact.^378^, ^379^, ^380^, ^381^

Modern medical technology has advanced biomaterials' ability to replace damaged tissue and organs while evanescing their functionality. One of the apatite minerals, HAp, makes up between 60 and 70 percent of the inorganic portion of the bone matrix and has a high ability to exchange ions with different cations, giving it high biocompatibility and bioactivity properties.^382^, ^383^, ^384^, ^385^ Bioactive materials, including HAp and other calcium phosphates, are better antibiotic delivery systems because they combine improved osteoconductive with drug transport capabilities.^386^

As a bioactive and biocompatible material, HAp can be an excellent option to apply on the surface of dental implants; however, because of its poor mechanical properties, which have limited its usage to non-load-bearing parts, its strength, roughness, and fracture toughness need to be improved for application in load-bearing parts.^387^, ^388^, ^389^ The Sol-gel method is highly appreciated for maintaining the characteristics of hydroxyapatite's bioactivity, as it regulates the phase purity, pH level, and chemical similarity.^390^ Moreover, the Sol-gel method applies to every shape, which can be appropriate for any surface. In the case of osseointegration, it shows hardness, a stable mechanical phenomenon to control characteristics.^391^ Hence, HAp must be kept in powder form to avoid variation due to adverse effects after production. In contrast to HAp composites on a microscale, it has shown evanesced mechanical and bioactivities.^392^, ^393^, ^394^ A synthetic biomaterial called HAp is receiving much attention in advanced complex tissue engineering. Its chemical makeup is similar to the mineral found in natural bone, and HA's bioactivity promotes the required bone rebuilding.^395^ We also provide an overview of how HAp and other inorganic nanomaterial composites can improve HAp's bioactivity and biocompatibility, a topic of contemporary study interest.^308^

## Applications of hydroxyapatite nanoparticles in dental implants

Hydroxyapatite (HA) nanoparticles have been the subject of extensive research to determine whether they might have prospective uses in dental treatment for several reasons, including their biocompatibility and resemblance to the mineral component of human bone.

Nanocomposites, nanoimpression materials, and nanoceramics are some of the nanomaterials that have the potential to play a significant role in the effective repair of teeth that have been damaged by disease, caries, missing teeth, or fractures.^396^ As advancements in nanotechnology impacted the domains of diagnostics, materials science, restorative dentistry, and oral surgery, the subject of nano dentistry began to take shape around the year 2000. The goal of nano dentistry is to provide patients with complete dental healthcare. Several oral disorders may be avoided or treated early on thanks to the development of increasingly sophisticated and precise diagnostic techniques. Among the many available nanomaterials, decaying, carious, missing, and broken teeth may be effectively restored using nanocomposites, nanoimpression materials, and nanoceramics.^397^ HA particles' properties significantly impacted the recruitment patterns of innate immune cells and the injection of cytokines. Larger particles or smooth, spherical particles of similar size did not cause a robust inflammatory response, but tiny, needle-shaped particles did. According to the results, the properties of hydroxyapatite particles control the recruitment of immune cells and the subsequent inflammatory response. This suggests that HA particle properties may be customized to control immunological responses brought on by biomaterial implantation.^398^

Here is a more comprehensive discussion of their uses:

### Optimizing osseointegration via surface coating

Dental implants commonly replace missing teeth in patients with partial or total tooth loss. Titanium dental implants are popular due to their biocompatibility, mechanical strength, corrosion resistance, and ability to integrate with bone (osseointegration). Ti-based implants often perform surface modifications to prevent failure.^399^, ^400^, ^401^, ^402^, ^403^ The surface structure of dental implants can influence bone metabolism. Studies have shown that rough surfaces promote bone cell differentiation, growth, attachment, and mineralization. Standard methods to create roughness on implant surfaces include acid etching, plasma spraying, sandblasting, and hydroxyapatite (HA) coating.^403^, ^404^ Utilizing hydroxyapatite nanoparticles as a surface coating for dental implants addresses several essential elements of implant success. The enhanced osseointegration and biocompatibility of dental implants coated with hydroxyapatite nanoparticles (HANP) are among the mentioned benefits. On the one hand, it doesn't provide precise information regarding patient outcomes, which might vary significantly from patient to patient based on factors like age, gender, systemic illnesses, or lifestyle choices like smoking. Although these factors are so important, younger, healthier people may have differing healing responses and implant success rates than older patients or those with systemic problems. The reaction to HANP-coated implants may vary widely.^405^, ^406^, ^407^ Additionally, lifestyle choices that hinder blood flow and healing, like smoking, might counteract the advantages of HANPs. If a correct understanding of the impact of HANP-coated dental implants is to be attained, it is necessary to examine the efficacy of these implants in various patient groups.^408^ These characteristics include mechanical stability, bioactivity for eHANPsnced osseointegration, and the implant's capacity to last for an extended period. Better patient results and a more dependable method of replacing lost teeth result from this.

#### Mechanical stability

Dental implants need a healthy and secure relationship with the bone surrounding them to maintain their effectiveness and longevity. An implant's capacity to endure the stresses of speaking and chewing depends on its mechanical stability.^409^ In the process of putting a coating on the surface of dental implants, hydroxyapatite (HA) nanoparticles are frequently used. This coating evanesces the implant's mechanical stability.^410^ A biomimetic surface that is more compatible with the bone surrounding it is created by incorporating a layer of hydroxyapatite (HA), a significant component of natural bone mineral.^411^ Hydroxyapatite nanoplates (HANPs) are more biocompatible, osteoconductive, and long-term stable than graphene and bioactive glasses for various reasons. By promoting cell adhesion, proliferation, and differentiation, HANPs have shown biocompatibility.^412^ They are also often less cytotoxic than some graphene derivatives. Their osteoconductivity is based on their capacity to promote cellular connections and bone formation due to their chemical resemblance to genuine bone. In some experiments, they often perform better than bioactive glasses. Long-term stability under physiological circumstances is shown by HANPs, which are more resilient to environmental deterioration than other materials, including bioactive glasses, and do not change chemically over time. The comparison side of this research shows certain potential benefits of HANPs for bone regeneration applications; however, further extensive testing using the newest biomaterial systems may show specific areas of higher relative performance.^413^, ^414^, ^415^

#### Bioactive interface

The interaction between the implant and the bone is vital in determining whether the implantation procedure will succeed.^416^ HA nanoparticles play an essential role in the development of a bioactive interface. This indicates that osteoblasts, the cells responsible for bone production, are actively encouraged to adhere to and multiply on the implant surface.^417^ Nanoparticle modification of dental implants has been explored for eHANPsnce osseointegration and mimicking the natural structures of the host tissue. Hydroxyapatite (HA) coatings have shown promising interactions with the biological environment, particularly in promoting the integration of osseous tissues with implant surfaces. HA coatings may serve as a calcium and phosphate ions source in a supersaturated state similar to enamel minerals. Additionally, HA coatings may support the remineralization of outer enamel caries lesions.^418^, ^419^ Bone-forming cells are stimulated to attach themselves to the implant surface by the bioactive interface that is produced by HA nanoparticles. The direct structural and functional bond between the implant and the bone is known as osseointegration, which can eHANPsnce this process. This leads to a smoother integration of the implant into the jawbone, which improves stability and reduces the possibility of implant failure.^420^

### Drug delivery

Hydroxyapatite nanoparticles have been the subject of intense research for their potential as drug delivery systems. A developed system uses HA nanoparticles grafted with Methotrexate (MTX) and Poly Vinyl Alcohol (PVA) through emulsion polymerization. These nanoparticles, loaded with Gemcitabine (GEM), an anti-cancer drug, have shown the ability to penetrate bones. In vitro studies of the drug release showed a combined release of approximately 25% after ten days, while physically loaded nanoparticles released around 60%. The sequential delivery of GEM followed by MTX was observed in vitro, suggesting increased potential for effective treatments and improved therapeutic efficiency. This research opens up exciting possibilities for the future of drug delivery in dentistry.^421^ Moreover, hydroxyapatite nanoparticles have potential applications in the oral environment. To develop a system for cell proliferation and bone regeneration in the oral cavity, Rajabnejadkeleshteri and colleagues synthesized Strontium Fluor-hydroxyapatite nanoparticles (F-SrHA). Fluorine was included to aid in the proliferation of osteoblasts, while strontium was chosen for its ability to improve gene expression in osteoblastic cells. The group utilized the precipitation technique and pH-cycling method to create these nanoparticles. Following cell incubation, the hydroxyapatite's added elements promoted cell growth and differentiation, suggesting potential dental applications.^422^

### Remineralization

Tooth decay^423^ and erosion^424^ occur when the hydroxyapatite minerals, the primary constituent of teeth, are dissolved due to acidic conditions. Compared to other biomineralized tissues like bone and dentin, mature enamel is acellular and does not undergo absorption or remodeling. The process of enamel formation, also known as amelogenesis, is meticulously regulated and involves precise genetic control, interactions between proteins, interactions between proteins and minerals, and interactions with membranes.^425^ As a result, it is not possible to regenerate enamel in vivo once it has failed. Currently, the most crucial and practical approach to prevent dental caries or acid erosion and restore the original structure of teeth is to inhibit tooth demineralization or promote tooth remineralization.^426^ Compared to other materials such as casein phosphopeptide calcium phosphate paste,^427^ bioactive glass, Casein Phosphopeptide-Amorphic Calcium Phosphate (CPP-ACP),^428^ amine fluoride and sodium monofluorophosphate,^429^ and fluoride varnish,^430^ hydroxyapatite possesses a superior capacity. Research has indicated that remineralization is associated with the physical deposition of hydroxyapatite crystals within the pores on the surface affected by demineralization.^431^

### Reconstructive dental care

The ideal luting agent would have to meet a long list of criteria, including biocompatibility, sufficient working time, flowability, compressive strength, minimal microleakage, low solubility in oral fluids, adhesiveness, aesthetics, affordability, and ease of removal in the event of excess material. Unfortunately, such an agent does not yet exist.^432^ In addition to emitting fluoride, Glass-Ionomer Cements (GICs) provide excellent adhesion to the enamel layer and dentin to a lesser degree.^433^ Researchers Kheur et al. evaluated traditional GIC, resin-modified GIC, and adhesive resin to determine the effects of adding HA NPs to glass-ionomer luting agents on the tooth's flexural and shear binding strengths. The experimental GIC modified with HA NPs was found to have superior bonding between the carboxyl groups of the polyacid with calcium from natural tooth structures to synthetic hydroxyapatite, despite adhesive resin having the highest flexural strength and shear bond strengths, according to the authors' conclusions.^433^

The findings of a comparable investigation conducted by Alatawi et al. (2018) demonstrated that introducing hydroxyapatite nanoparticles (HA NPs) into conventional glass-ionomer cement increased the discharge of fluoride ions. Furthermore, an 8% nano-HA concentration resulted in considerable bacterial suppression against S. Mutans.^434^ When comparing human Dental Pulp Stem Cells (DPSCs) to a standard GIC and a resin-modified GIC, Noorani et al. assessed the cytotoxic effects of nano-hydroxyapatite–silica incorporated into GIC. The study's nano-hydroxyapatite-silica-glass-ionomer cement outperformed the resin-modified GIC in terms of biocompatibility and cytotoxicity when compared to the regular GIC.^435^ The survey conducted by Pagano et al. examined a hybrid product of greater complexity. Mechanical, thermal, and biological characteristics of glass-ionomer cement—a nano-hydroxyapatite, mucosal defense agent, and antibiotic emulsion—were synthesized and assessed. The study's null hypothesis was that the new product would not alter the mechanical, thermal, or microbiological characteristics of GIC, nor their levels of cytotoxicity. The outcomes demonstrated improved mechanical qualities, especially with the powder-formulated antibiotic and in a moist environment like the oral cavity. HA NPs, an antibiotic and a mucosal defense agent, significantly decreased the total cytotoxicity of the cement.^436^ Juntaveeet al. devised a compelling approach through their investigation into the impact of Clinpro (CP) and nano-hydroxyapatite gel on the remineralization capacity of cementum and enamel at the cavo surface area of ceramic restorations manufactured and designed with computer-aided technology. Their study showed that, in comparison to non-treated demineralized surfaces, both HA NP gel and CP are significantly capable of remineralizing to aid in the recovery of demineralized enamel and cementum in a clinical context where irregularities at the cavosurface junction and micrographs at the tooth–restoration interfaces are always present and induce bacterial accumulation leading to tooth decay.^437^

## Current challenges and future opportunities

Despite the enthusiasm around the rapid advancement of nanotechnology applications, evaluating possible risks connected to exposure to nanoscale materials and their results has become a crucial topic of research in toxicology and health risk assessment. It is somewhat paradoxical that the same unique qualities that make nanoscale materials helpful also make them potentially dangerous in some circumstances.^438^ This review introduces novel work on hydroxyapatite nanoparticles (HANPs) that emphasizes its use in dental implant applications. In contrast to most previous investigations, this article has introduced new synthetic methods intended to enhance the osseointegration, structural characteristics, and biocompatibility of HANPs. It also looks at the benefits of HANPs outside of implant applications, such as desensitization in mouthwash and antimicrobial coatings, and as part of a thorough integration of HANPs in contemporary dentistry treatments.^439^ There are still many more to overcome to overcome current challenges and prospects.

### Current challenges

Studies on the biocompatibility of HAp have been reported often in recent years. For instance, Remya et al. evaluated the molecular toxicity of 50 nm HAPs utilizing Bone Marrow Mesenchymal Stem Cells (BMSCs), and they concluded that the internally produced HAPs were neither poisonous nor unsafe to BMSCs.^440^ Wang et al. investigated the toxicity of HAPs on the principal organs of SD rats and MC3T3-E1 cells in vitro. They found that HAPs might cause cell death in the liver and renal tissues and decrease MC3T3-E1 cell growth.^441^ Further research was done by RSC Adv in 2017 on the cytotoxicity of HAPs on MC3T3-E1 cells and the associated processes. The findings demonstrated that HAp could enter MC3T3-E1 cells by an endocytosis pathway driven by macropinocytosis and were primarily located in lysosomes. They conducted an annexin V-FITC/PI apoptosis test, which revealed that HAp dramatically increased the rate of cell death, and the mechanism underlying this increase was connected to oxidative stress.^442^ HAp's most significant limitation is its limited strength, making it challenging to produce high-load-bearing implants from Hap.^443^ The main concern during the application of HAp coatings is the need for HAp to adhere more to the metallic surface. This is due to a poor adhesive bond between the HAp layer and the metallic load-bearing areas.^444^ Surface modifying chemicals are required to increase HAp films' adhesion. These substances assist in forming a long-lasting coating on top of the metallic surface, aiding in achieving the intended result. Over the past 50 years, HAp, the main inorganic component of hard tissues (bones), has been utilized in several biomedical applications due to its biocompatibility. On the other hand, a previously conducted and published study discovered that HAp exhibits the traits of brittle ceramics that cannot withstand the weight of a load.^445^ In addition, the implants in use have deteriorated after several years, which is attributable to their poor wear and corrosion resistance. The occurrence of porosity in HAp-coated implants hurts the implants' ability to resist corrosion in a physiological environment.^446^ However, in some specific applications, selecting any particular approach to synthesize a well-defined powder might be difficult because there are many ways to manufacture HAp nanoparticles.^447^ According to experts, modifying the structural properties of currently existing biocompatible and biodegradable materials is the most crucial topic that still needs particular attention and will define the future course of research in this field.^448^

### Future opportunities

With the ongoing advancements in nanotechnology and materials science, new materials are created for various purposes.^449^ The use of hydroxyapatite nanorods to mimic the natural biomineralization process that forms dental tissue enamel was described by Clarkson et al.^450^; they modified the surface of the nanorods by depositing a monolayer of surfactant to enable them to assemble into an enamel structure resembling a prism at the water/air interface—additionally, the same category of applications for enamel is in this line. The ability of hydroxyapatite nanorods to be reinforced with polyethylene, as shown by Mangalaraj and colleagues,^451^ may be advantageous when utilized as tissue scaffolds and reinforcement materials. Using a hydrothermal process, Atai et al.^452^ created hydroxyapatite nanorods that were then integrated into a dentin adhesive. They noted that the nanorods were stable in the solution and evenly spread, preventing particle aggregation. They also pointed out that the nanorod-doped glue had increased bioactivity. Hydroxyapatite nanoparticles (HANPs) have been shown to enhance osseointegration generally, especially in individuals with reduced bone density, such as osteoporosis sufferers and the elderly. HANPs enhance bone cells' biological response, improving bone remodeling and excellent adhesion to dental implants. The reduced trabecular bone density seen in various patient groups may be overcome with these treatments.^453^

Hydroxyapatite may create problems with bacteria present in saliva for long-term usage. HAp normally doesn't react adversely with biofilms within 48 hours, even with saliva.^454^ However, disaggregated HAp can be compelling in long-term reactions with biofilms. Researchers have already shown a biofilm model for bacterial growth where Sodium Hexametaphosphate (SHMP) is used to disaggregate HAp NPs for long-term benefits of use in an oral environment.^455^ Additionally, some oral bacteria may have adverse effects on coating HAp nanoparticles. However, modern research shows some positivity in oral bacteria with the coexistence of HAp NPs. Adding a catalyst with HAp can change the positive reaction with oral bacteria. Table 8 shows some references to it.

**Table 8:** Behavior of some oral bacteria with hydroxyapatite.

Oral Bacteria	Catalyst	Behavior with Hydroxyapatite	Ref.
Streptococcus	ZnO	Provide osteoconductive and antimicrobial functionalities	^456^
Streptococcus mutans	TiO2	Accelerate of enamel mineralization	^457^
Lactobacillus acidophilus	SiO2	Offer antimicrobial qualities to combat mouth infections.	^458^
Peptostreptococcus	Antimicrobial peptides	Killing bacteria directly	^459^

However, researchers have created a perfect material with the same elasticity, toughness, and mechanical strength as genuine bone by joining polymers with calcium phosphate and HA in various ways. The research should focus on the bio-applications of stiff and flexible polymers with HA-NMs bio-interactive interface. Because they are more malleable and have more strength, HA-NMs with hard and soft polymers may be employed for bone replacement and skin tissues, respectively.^460^ By substituting carbonate at phosphate and hydroxide sites in Biological Apatites (BAp), such as enamel, dentine, bone, and pathological calcifications, it tends to improve their solubility compared to pure HAp. An experiment was conducted where only a minor carbonate was found in the end-point solid at pH 7.4 and pCO_2_ = 0.011.0 bar. Further research is necessary to fully understand the ramifications of these findings since they relate to a crucial area of oral chemistry.^461^ A plausible reason for this was recently discovered by Tang and colleagues.^462^ They employed significantly smaller (20 nm) hydroxyapatite particles in their research, which match in size far superior to dental enamel's native hydroxyapatite. These investigations are encouraging, particularly considering combining this strategy with a resin-based restorative dental material. The ability to repair enamel in vivo and show that the regenerated enamel is mechanically stable will be crucial in the future.^463^ So, the following recommendations can be provided for better research-

• Implement more sophisticated hydrothermal and sol-gel methods for synthesis.
• Examine the variation of metals used to make adhesives with HAp to form dentine.
• Improve mechanical characteristics such as hardness and flexibility in fabrication HAp in teeth.
• Implementing more clinical trials to confront the ability of HAp NPs for medical technology

## Conclusion

Modern dentistry benefits from cutting-edge technology, with Hydroxyapatite Nanoparticles (HA NPs) particularly noteworthy for their innovative properties. These nanoparticles contribute to the strength and rigidity of both teeth and bones. Their properties closely resemble natural dentin and enamel, making them suitable for dental implants and treatments. Additionally, HA NPs promote relentless tooth regeneration without provoking any adverse reactions, offering solutions for tooth decay, root canals, accidental damage, and cavities. Furthermore, these nanoparticles form a strong bond with natural organs such as bones and tissues, facilitating drug transportation during dental implants and other dental treatments.

Hydroxyapatite Nanoparticles play a crucial role in preventing cavities and evanescing teeth whitening. Additionally, they effectively treat dentin hypersensitivity by facilitating chemical reactions that alleviate the patient's discomfort. These nanoparticles also aid in implant procedures, acting as a catalyst for the implantation techniques. Post-implantation, Hydroxyapatite nanoparticles promote the remineralization and accelerate the osseointegration process. Due to their advanced biocompatibility, HANPs foster favorable biological environments on the surface and inside the teeth. In dentine formation, HAp is utilized in conjunction with pulp capping techniques. Its preventative attributes contribute to improved tissue adhesion, thereby minimizing adverse effects.

Future research is expected to confirm further the impact and superiority of HA NPs on dental replacement. Hydroxyapatite is anticipated to continue to provide a higher level of sophistication for surface modification by ensuring a biological environment and corrosion resistance. Several characterization techniques, such as SEM, FE-SEM, EDX, etc., are believed to continue to shed light on the suitable purity, shape, dispersion, and strength required for implantation. Specific fabrication processes will likely be applied based on future requirements to achieve the desired results. Although further research is needed to confirm the effects, the biocompatibility behavior is anticipated to continue to be validated. It is also expected that drug delivery systems will continue to outperform other oral treatments, and toxicity levels will need to be consistently controlled. Anticipations suggest that in vivo and in vitro studies will continue to show extensive possibilities for strengthening enamel formation in dental regeneration through Hydroxyapatite.

## Discussion

This paper mainly focuses on the synthesis techniques, their pros and cons, and the potential uses of HANPs in dental. Enhancement in the antibacterial, osseointegration, and biocompatibility qualities should be discussed. This portion might also benefit from comparing with issues like phase purity and scalability and their ensuing implications for future use in orthopedic and dental implants. Evaluatingthe general theory of dental implants that include hydroxyapatite nanoparticles (HANPs) may significantly increase efficacy by enhancing osseointegration, encouraging bone growth, and providing antimicrobial qualities, all of which improve patient outcomes. Dental implant technology may be transformed from materials to patient care by these fantastic biocompatible HANPs. Additionally, this review showed the therapeutic importance of hydroxyapatite nanoparticles (HANPs) in dental implants and their osteoconductive and highly biocompatible qualities. These particles improve implant integration in bone, aid bone development, and encourage bone growth since they resemble natural bone structures. As a result, HANPs have been clinically shown to alleviate dentinal hypersensitivity and aid in preventing the development of germs on implants.

## Conflict of interest

The authors declare that they have no known competing financial interests or personal relationships that could have appeared to influence the work reported in this paper.
